# Citrullination of Histone H3 Interferes with HP1-Mediated Transcriptional Repression

**DOI:** 10.1371/journal.pgen.1002934

**Published:** 2012-09-13

**Authors:** Priyanka Sharma, Saliha Azebi, Patrick England, Tove Christensen, Anné Møller-Larsen, Thor Petersen, Eric Batsché, Christian Muchardt

**Affiliations:** 1 Institut Pasteur, Département de Biologie du Développement, CNRS URA2578, Unité de Régulation Epigénétique, Paris, France; 2 Institut Pasteur, Département de Biologie Structurale et Chimie, CNRS UMR3528, Plate-Forme de Biophysique des Macromolécules et de Leurs Interactions, Paris, France; 3 Department of Biomedicine, Aarhus University, Aarhus, Denmark; 4 Department of Neurology, Aarhus University Hospital, Aarhus, Denmark; Duke University, United States of America

## Abstract

Multiple Sclerosis (MS) is an autoimmune disease associated with abnormal expression of a subset of cytokines, resulting in inappropriate T-lymphocyte activation and uncontrolled immune response. A key issue in the field is the need to understand why these cytokines are transcriptionally activated in the patients. Here, we have examined several transcription units subject to pathological reactivation in MS, including the *TNFα* and *IL8* cytokine genes and also several Human Endogenous RetroViruses (HERVs). We find that both the immune genes and the HERVs require the heterochromatin protein HP1α for their transcriptional repression. We further show that the Peptidylarginine Deiminase 4 (PADI4), an enzyme with a suspected role in MS, weakens the binding of HP1α to tri-methylated histone H3 lysine 9 by citrullinating histone H3 arginine 8. The resulting de-repression of both cytokines and HERVs can be reversed with the PADI-inhibitor Cl-amidine. Finally, we show that in peripheral blood mononuclear cells (PBMCs) from MS patients, the promoters of *TNFα*, and several HERVs share a deficit in HP1α recruitment and an augmented accumulation of histone H3 with a double citrulline 8 tri-methyl lysine 9 modifications. Thus, our study provides compelling evidence that HP1α and PADI4 are regulators of both immune genes and HERVs, and that multiple events of transcriptional reactivation in MS patients can be explained by the deficiency of a single mechanism of gene silencing.

## Author Summary

In patients suffering from Multiple Sclerosis (MS), T lymphocytes express abnormally high levels of a subset of cytokines. The same cells also transcribe a series of vestigial retroviral sequences normally silenced by chromatin factors. In this study, we have searched for regulatory mechanisms shared between the cytokines and the retroviral sequences. We find that the repressor protein HP1α is present on the promoter of both types of transcription units in normal cells and that the recruitment of this protein to these promoters is decreased in MS patients. Furthermore, we show that the delocalization of HP1α from these promoters can be caused by citrullination of histone H3, and we provide evidence indicating that levels of this histone modification is augmented in MS patients. Together our data provide a possible explanation for the simultaneously increased transcriptional activity of cytokines and endogenous retroviruses in MS-patient T lymphocytes and suggest that inhibitors of the enzyme responsible for the increased citrullination of histone H3 could help restore normal levels of cytokine activity in the patients.

## Introduction

Multiple Sclerosis (MS) is a progressive inflammatory disease of the central nervous system in which leukocytes and antibodies attack myelin sheaths, resulting in demyelination and ultimately destruction of the axons [1]. Many lines of evidence point at inappropriate activation of T cells as an initiating event of the pathological process, although the mechanism at the root of this T cell activation is still poorly defined (for a recent review see [2]).

In MS patients, activation of the T cell population is associated with increased expression of a series of cytokines [3], [4]. The abnormally abundant expression of the genes encoding these regulators of the immune system may be a consequence, but also possibly a cause of the activation of the T cells. It is therefore essential to explore the mechanisms that keep these genes in check in normal cells and that may be defective in MS patients.

Interestingly, in MS and other autoimmune diseases including Rheumatoid Arthritis and Systemic Lupus Erythematosus, transcription of Human Endogenous RetroViruses (HERVs) is also increased in T cells [5], [6], [7]. HERVs are abundant vestigial retroviral sequences that in healthy cells are largely silenced by the epigenetic mechanisms repressing most repeated DNA sequences. These mechanisms include DNA methylation and histone H3 lysine 9 (H3K9) methylation. DNA methylation favors chromatin compaction by promoting recruitment of histone deacetylases (HDACs) or alternatively by directly reducing the affinity of transcription factors to their cognate DNA binding sites [8]. Consistent with this, deletion of the *de novo* DNA methyltransferase Dnmt1 results in massive re-expression of ERVs in the mouse embryo [9]. DNA methylation at ERV promoters is particularly high in differentiated mouse cells [10], while it may be partially dispensable in mouse embryonic stem (ES) cells [11].

In these cells, the major source of silencing appears to be H3K9 methylation [12], [13], [14]. This histone modification is recognized by a number of proteins containing either chromo- [15], [16], [17], [18], [19], [20], MBT- [21], PHD- [22], or Tudor- [21], [23] domains. Mainly HP1 proteins have been detected on mouse ERV promoter sequences [24], [25], although their role in the repression of these sequences in mouse ES cells is still at debate [26].

HP1 proteins are particularly interesting in the context of MS because in addition to their possible function in the silencing of repeated DNA [27], [28], they are present on the promoters of a number of genes involved in immune defense, including the immunomodulatory cytokine *TNFα* [29], the interleukins *IL1β* [30], [31], *IL6* [32], and *IL8* [33], and several interferon-inducible genes [34]. They also participate in the regulation of the HIV1 long terminal repeat (LTR) that shares several regulatory mechanisms with immune genes [35], [36], [37].

Consistent with their role in the transcriptional control of inducible genes, the binding of HP1 proteins to chromatin is subject to regulation. In particular, the methylation mark on histone H3 can be removed by histone demethylases [38]. A more transient regulation of HP1 binding may occur by modification of residues neighboring H3K9, including phosphorylation of serine 10 and acetylation of lysine 14 [39], [40], [41]. The histone H3 arginine 8 (H3R8) located immediately upstream of H3K9, is also subject to modifications [42] that theoretically could interfere with HP1 binding, although this has never been investigated. This arginine can be either methylated [43] or converted into the non-coding amino acid citrulline [44], [45], [46], [47], [48].

Citrullination of H3R8 is catalyzed by the calcium-dependent peptidylarginine deiminase PADI4. This enzyme, that is the only member of its family to enter the nucleus, also citrullinates histone H3 on arginines 2, 17, and 26, as well as histones H2A and H4 on their respective arginine 3 [44], [45], [46], [49]. Many reports describe PADI4 as a regulator of transcription. On p53 targets [50], [51], and estrogen-regulated genes, including *pS2* [44], [45], [52], it functions as a repressor either by interfering with activating arginine-methylation events [44], or by favoring recruitment of HDACs [52]. Inversely, PADI4 also associates with a number of transcriptionally active promoters and functions as an activator of *c-Fos via* a mechanism that involves facilitated phosphorylation of the ETS-domain protein Elk-1 [53]. In Asians, hyperactivity of PADI4 has been associated with Rheumatoid Arthritis [54], [55], [56], and contributes to the generation of antibodies directed against citrullinated proteins during the development of this disease [57]. An earlier study has also reported increased nuclear localization of this enzyme in white brain matter of MS patients [58]

The transcriptional deregulation of both cytokines and HERVs in MS patient T cells prompted us to investigate a possibly co-regulation of these two types of transcription units by HP1 proteins. At cytokine genes and HERVs we examined in tissue culture cells, transcriptional repression required HP1α, while PADI4 functioned as an activator by destroying the HP1α binding site on the tail of histone H3. Consistent with this, we observed that in circulating blood cells from MS patients, recruitment of HP1α to the promoter of the master cytokine TNFα and to HERV sequences is significantly reduced, while citrullination of H3R8 at these positions is increased. Taken together, our data strongly suggest that increased citrullination of histone H3 can antagonize gene-specific chromatin-mediated silencing in T cells and thereby participate in increased cytokine expression during the normal inflammatory response and in MS patients.

## Results

### The transcriptional regulator HP1α is shared by HERVs and cytokines

**Figure 1:**
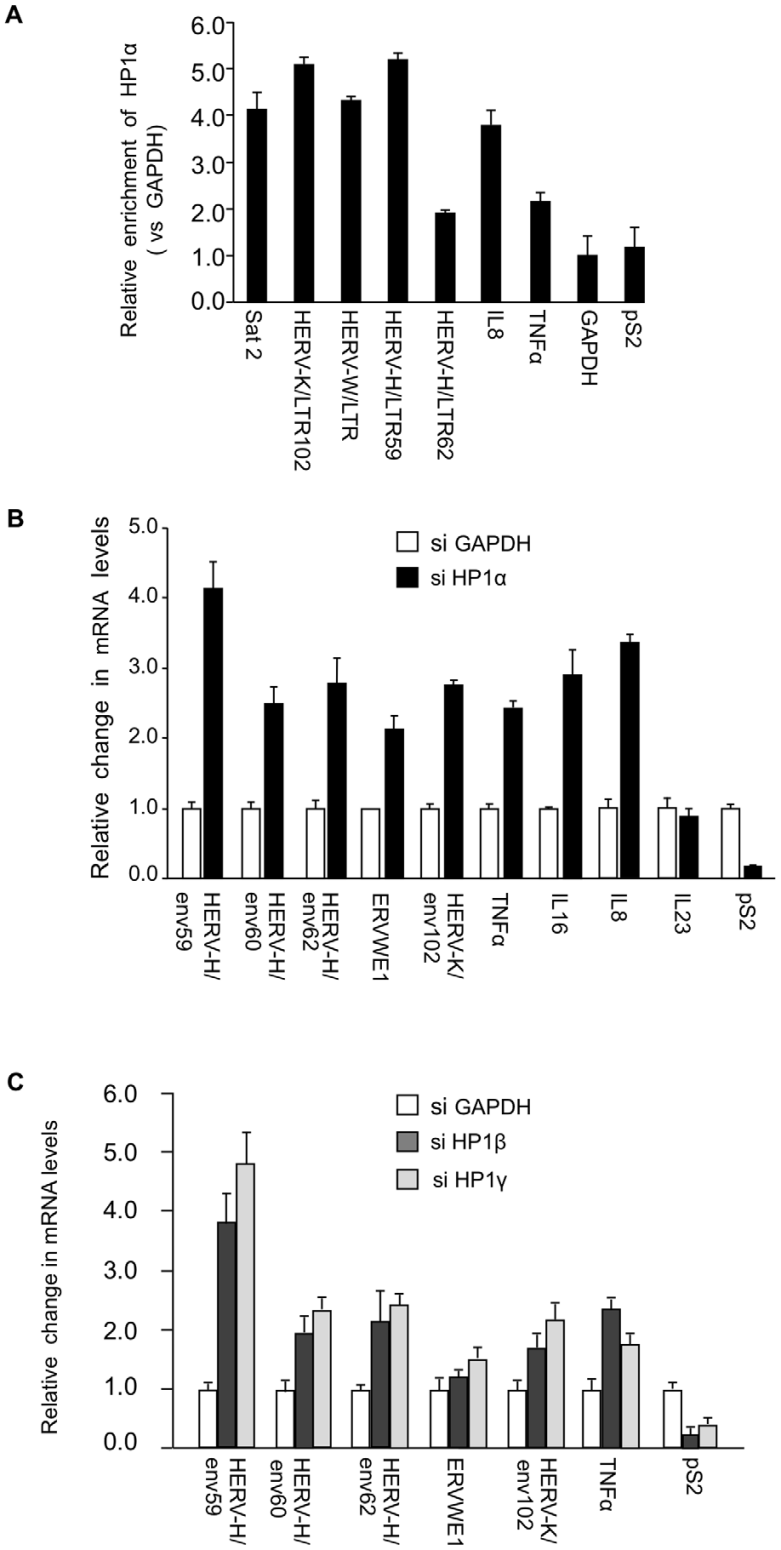
HP1α involved in silencing of HERVs and cytokines. (A) HP1α is present on the promoter of HERVs and cytokines in MCF7 cells. Chromatin prepared from MCF7 cells were subjected to ChIP using anti-HP1α antibodies. The relative enrichment was measured by qPCR on Satellite 2 sequences, the LTR regions of shown HERVs and on the promoter regions of *TNFα*, *pS2* and *GAPDH*. Data are normalized to the values obtained with non-immune IgGs and expressed relatively to *GAPDH* (set to 1). Shown data are means +/− SEM from four experiments. (B and C) Total RNA from MCF7 cells transfected with the indicated small interfering RNAs (siRNAs) was quantified with RT-qPCR. Changes in mRNA levels are shown relative to the siGAPDH transfection (set to 1), which was not affecting the mRNA levels of the genes of interest (Figure. S1B). The data are presented as the means ± SEM of triplicate experiments.

While several reports describe an implication of HP1 proteins in the regulation of genes involved in immune defense [29], [30], [31], [32], [33], [34], the role of these proteins in the silencing of HERVs in human cells needed to be clarified. We carried out these experiments in MCF7 cells, a breast tumor-derived cell line frequently used to examine expression of HERVs (see for example [59]). Chromatin Immunoprecipitation (ChIP) assays demonstrated that in these cells, HP1α accumulates on HERV-K, HERV-W, and HERV-H promoters at levels similar to those observed on Satellite-2 sequences (Figure 1A). As expected, HP1α was also detected on the promoters of cytokines TNFα and IL8. Consistent with this, depletion of HP1α with small interfering RNAs (siRNAs) resulted in increased expression of the HERVH/env62, HERVH/env59, HERVH/env60, ERVWE1, HERVK/env102, TNFα, IL8, and IL16, a cytokine also relevant for MS [60] (Figure 1B, Figure S1A and S1B). In these experiments, expression of IL23 [61] was unaffected, while the control estrogen-responsive *pS2* gene was repressed rather than activated. Reactivation of HERVs and TNFα was also observed upon depletion of HP1β and HP1γ, two other members of the HP1 family (Figure 1C).

### PADI4 citrullinates the binding site of HP1α on histone H3 *in vivo*

**Figure 2:**
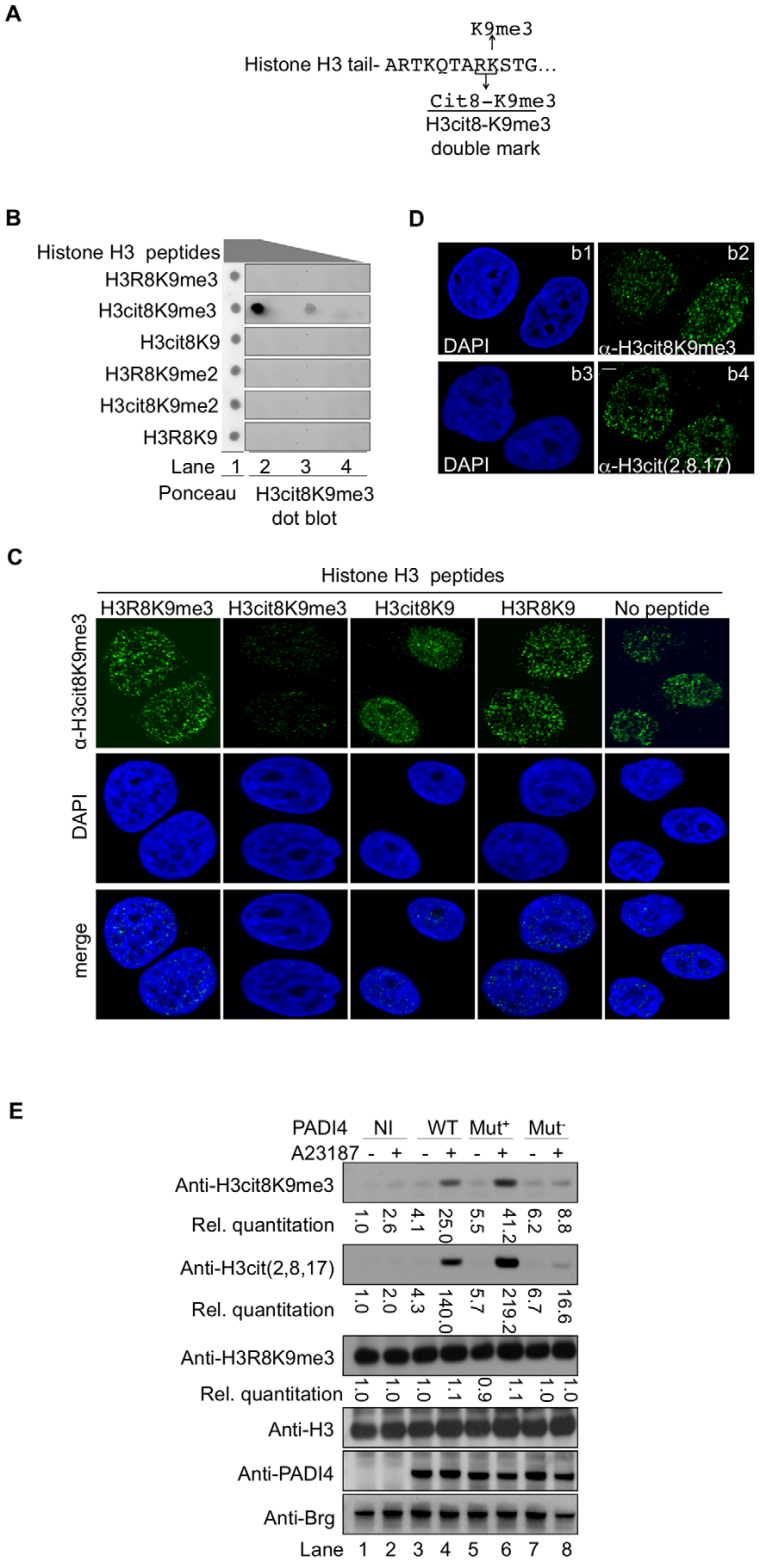
An antibody specific for histone H3cit8K9me3 reveals increased levels of this double modification catalyzed by PADI4. (A) Epitope used to generate the anti-H3cit8K9me3 antibody. The antibody was raised against a peptide mimicking histone H3 citrullinated at position 8 and tri-methylated on K9. (B) Dot blot highlighting the specificity of the anti-H3cit8K9me3, performed with 1, 0.2, and 0.04 µg of H3 peptides bearing the indicated post-translational modifications. (C) Specificity of the anti-H3cit8K9me3 antibody test by competition with H3 peptides. MCF7 cells were extracted, fixed and incubated with the indicated peptides along with anti-H3cit8K9me3 antibodies and DAPI. Scale bar 5 µm. (D) Similar staining with anti-H3cit8K9me3 and anti-H3cit(2,8,17) antibodies. Indirect immunofluorescent staining of MCF7 cells with DAPI to visualize the DNA and either anti-H3cit8K9me3 (b1–b2) or anti-H3cit(2,8,17) (b3–b4). (E) HEK293 cells that stably integrate a ponasterone A-inducible construct with either no insert (NI) or an insert coding for wild-type (WT), hyperactive (Mut+), or hypoactive (Mut−) PADI4 were cultured without or with ionophore (A23187). Total protein extracts were immunoblotted with the indicated antibodies. Signals were quantified with the Image J software (NI without treatment set to 1).

HP1α binds tri-methylated histone H3 lysine 9 (H3K9me3). The neighboring H3R8 residue is one of the 3 arginines recognized by the anti-H3cit (2, 8, 17) antibody used to show increased histone H3 citrullination in MS patients [58]. This raised a possibility of interference between citrullination of H3R8 and HP1α binding to histone H3. We therefore explored whether citrullination of histone H3R8 occurs *in vivo* on histone H3 tails already tri-methylated on K9. To this end, we generated an antibody recognizing the double citrullination-methylation modification H3cit8K9me3 (Figure 2A).

The specificity of this antibody was verified by dot blots using synthetic peptides mimicking modified histone tails (Figure 2B) and by testing the ability of the same peptides to compete with the binding of the antibody to cellular targets in fixed breast cancer-derived MCF7 cells (Figure 2C). In the later assay, the anti-H3cit8K9me3 antibody yielded an immunofluorescent staining very similar to that obtained with anti-H3cit(2,8,17) antibody (Figure 2D).

We next focused our attention on PADI4, the partially nuclear peptidylarginine deiminase responsible for the citrullination of histone H3. To determine whether this enzyme can citrullinate H3R8 when H3K9 is methylated, we generated HEK293-derived cell lines expressing either wild-type (WT), hyperactive [62], or hypoactive [63] versions of PADI4 under the control of a ponasterone-inducible promoter (Figure 2E). Induction of PADI4 synthesis and activity with ponasterone and the ionophore A23187, respectively, allowed detection of the H3cit8K9me3 double modification when the cells were expressing WT or hyperactive versions of PADI4. Under these conditions, we also observed a general increase in histone H3 citrullination, but no change in the levels of H3K9me3, indicating that H3R8 citrullination and H3K9 tri-methylation are not antagonistic.

Taken together, these experiments demonstrate that the H3cit8K9me3 double modification exists *in vivo* and that its formation is favored by increased PADI4 activity.

### Citrullination of H3R8 inhibits binding of HP1α to the histone H3 tail

**Figure 3:**
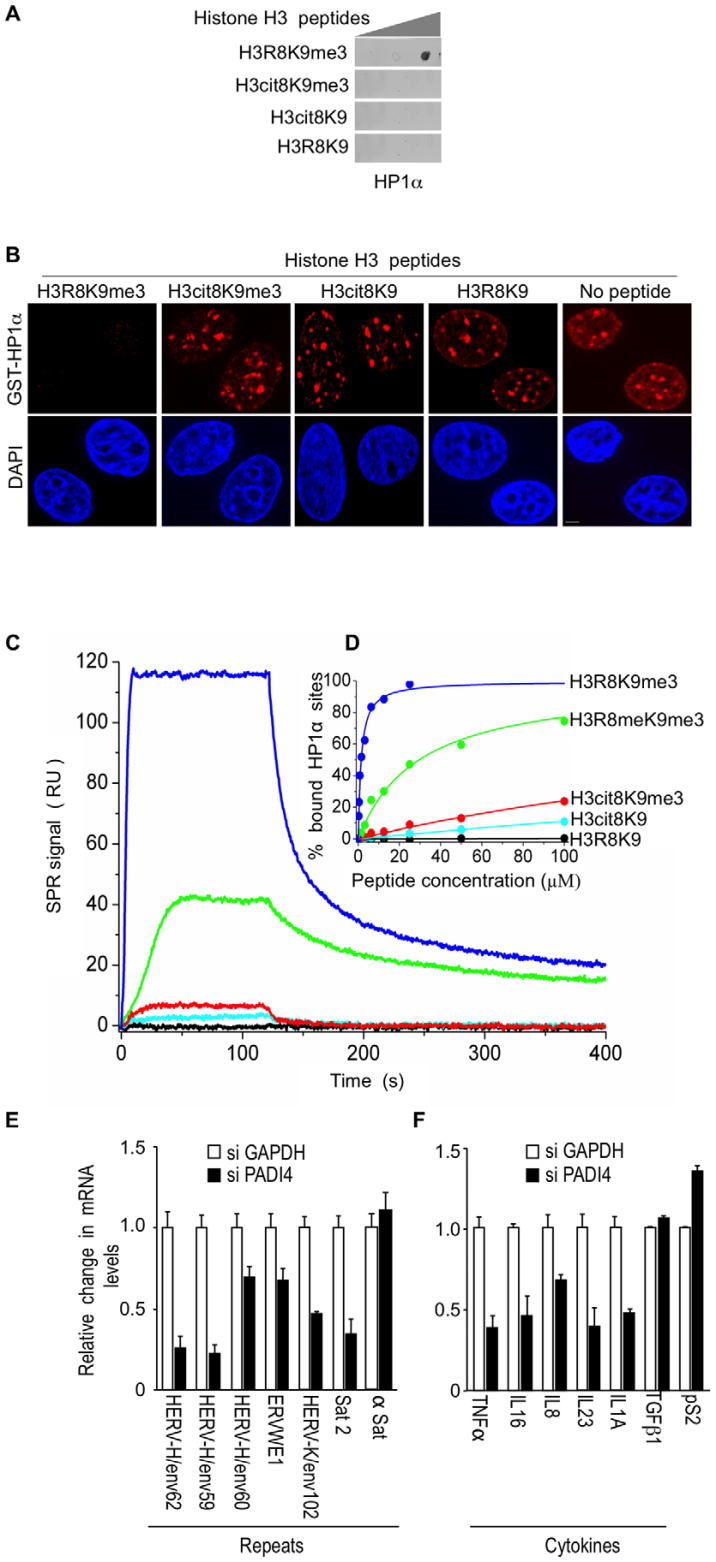
The H3cit8K9me3 double histone modification reduces the affinity of HP1α for the H3K9me3 single modification. (A) Dot blot showing that HP1α does not bind to histone H3 peptides carrying a H3Cit8K9me3 double modification. Indicated histone H3 peptides of 1, 0.2, and 0.04 µg were spotted on the membranes. Then binding of recombinant GST-HP1α was tested by labeling of the retained protein with anti-GST antibodies. Blots are representative of the experimental replicates. (B) Purified recombinant GST-HP1α (red) was bound to fixed MCF7 cells in “overlay” assays, and then challenged by competition with 1 µg of the indicated peptides. DNA is labeled with DAPI (blue), scale bar: 5 µm. (C) Real-time association and dissociation surface plasmon resonance (SPR) profiles corresponding to the injection of the indicated H3 peptides at 12.5 µM over immobilized GST-HP1α. (D) Percentage of bound GST-HP1α sites as a function of the peptide concentration. (E–F) Total RNA from MCF7 cells transfected with the PADI4 small interfering RNAs (siRNAs) was quantified with RT-qPCR. Changes in mRNA levels are shown relative to the siGAPDH transfection (set to 1), which was not affecting the mRNA levels of the genes of interest (Figure S1C). The data are presented as the means ± SEM of triplicate experiments.

We next investigate the impact of the H3R8 citrullination on the binding of HP1α to histone H3 tails tri-methylated on K9. When histone H3 peptides were spotted on membrane, HP1α bound a peptide carrying the single K9me3 modification, but not a peptide with a double cit8K9me3 modification (Figure 3A). We also tested the ability of these peptides to interfere with the binding of HP1α to its endogenous target sites. For this, we took advantage of the fact that recombinant GST-HP1α protein incubated on fixed permeabilized cells distributes in a pattern indistinguishable from that of the endogenous protein [64]. In this assay, while H3R8K9me3 peptide competed with the cellular sites for GST-HP1α binding, H3cit8K9me3 peptide did not (Figure 3B). As expected, H3cit8K9 and H3R8K9 peptides also failed to compete for GST-HP1α binding.

Surface plasmon resonance allowed us to quantify the effect of H3R8 citrullination and indicated a more than 200-fold decrease in the affinity of HP1α for H3cit8K9me3 compared to H3R8K9me3 (K_d_ 313±28 µM and 1.39±0.06 µM, respectively; Figure 3C–3D). We noted also that citrullination of H3R8 exerted a 10-fold higher effect on HP1α binding than did its methylation (K_d_ 29.6±1.3 µM).

To document that citrullination compromises transcriptional repression of HERVs and cytokines, we finally used siRNAs against PADI4 in the MCF7 cells known to express relatively high levels of PADI4 [65]. Depletion of this protein had an effect inverse to that of HP1α depletion and resulted in decreased levels of HERV transcripts (Figure 3E, Figure S1A and S1C). Levels of Satellite 2 transcripts (but not α-Satellite transcripts) were also decreased, suggesting a broad yet selective effect of citrullination on the silencing of repeats. PADI4 depletion also decreased expression of the immune genes *TNFα*, *IL16*, and *IL8*, as well as *IL23* and *IL1A*, but not *TGFß1* (Figure 3F). The control *pS2* gene known to be negatively regulated by PADI4 [44], [45], was, as expected, moderately stimulated.

### Endogenous PADI activity can be artificially regulated and directly controls HP1α-mediated repression

**Figure 4:**
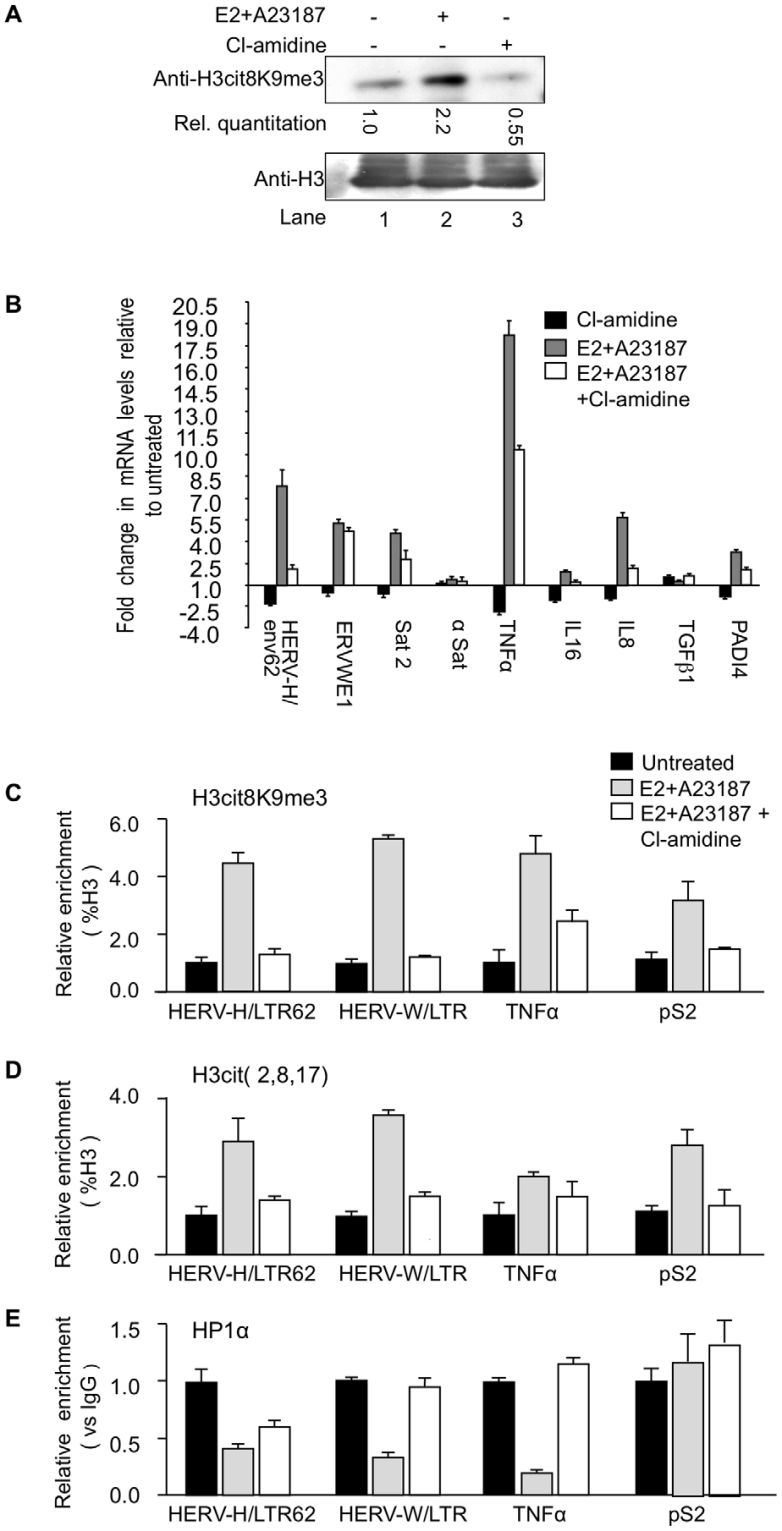
PADI4 activity controls HP1α occupancy and histone H3 citrullination at TNFα and HERV promoters. (A) Total protein extracts from MCF7 cells treated either estradiol (E2) or ionophore (A23187) and/or Cl-amidine were immunoblotted with the indicated antibodies. (B) Total RNA from MCF7 cells either untreated or treated with estradiol followed by ionophore (E2+A23187) and/or Cl-amidine (E2+A23187+Cl-amidine) treatment was quantified by RT-qPCR. Data are shown relative to the un-induced condition (set to 1). Values are mean ± SEM from four experimental replicates. (C–E) ChIP with the listed antibodies was carried out with chromatin from MCF7 cells either untreated or treated with estradiol and ionophore (E2+A23187) in the absence or presence of Cl-amidine. The relative enrichments of the indicated antibodies on the shown LTRs or promoters were measured by qPCR. Data are presented as a percentage of histone H3 or relative to non-immune IgG as indicated. Enrichments are presented relative to indicated controls (set to 1). Values are means ± SEM from four PCR measures of representative ChIP experiments.

MCF7 cells are estrogen-responsive and an estradiol (E2) treatment combined with an ionophore increases total levels of both PADI4 (Figure S2A and [65]) and H3cit8K9me3 modification (Figure 4A, compare lanes 1 and 2). In contrast, reduced PADI activity can be obtained with the specific inhibitor Cl-amidine [66]. This drug affects the nuclear PADI4 as illustrated by the decreased levels of H3Cit8K9me3 in MCF7 cells (Figure 4A; compare lanes 1 and 3). Thus, treatment with either E2/A23187 or Cl-amidine allowed us to control endogenous nuclear PADI activity at will.

As in the PADI4-depletion experiments, treatment of the MCF7 cells with Cl-amidine reduced expression of HER V-H/env62, ERVWE1, and the selected cytokine genes, but not α-Satellite and *TGFβ1* (Figure 4B, black bars). Inversely, augmenting PADI4 activity by treating the MCF7 cells with E2 and ionophore resulted in a Cl-amidine-sensitive increase in expression of the same HERVs and cytokines (Figure 4B, grey and white bars & S2B).

Finally, we performed ChIP to follow the impact of endogenous nuclear PADI activity on the citrullination of histone H3 and the recruitment of HP1α to the LTRs of HERV-H and ERVWE1 (HERV-W/LTR) and the promoter of *TNFα*. These assays confirmed that stimulation of PADI4 activity with E2 and ionophore locally increases levels of citrullinated histone H3, as detected with either anti-H3cit (2, 8, 17) or anti-H3cit8K9me3 antibody (Figure 4C–4D and Figure S2C), while the levels of HP1α recruitment were markedly decreased (Figure 4E, black and grey bars). These levels of HP1α occupancy were partially restored by further treating the cells with Cl-amidine (Figure 4E, white bars), illustrating that this inhibitor can overcome the detrimental effect of excessive PADI activity. The level of HP1α recruitment at the pS2 control promoter region was low and was not substantially affected by changes in citrullination levels (Figure 4C–4E).

Taken together, these results demonstrate that HP1α and citrullination antagonistically regulate several immune genes and HERVs, and that this regulation is druggable.

### PADI activity is required for normal activation of T cells

**Figure 5:**
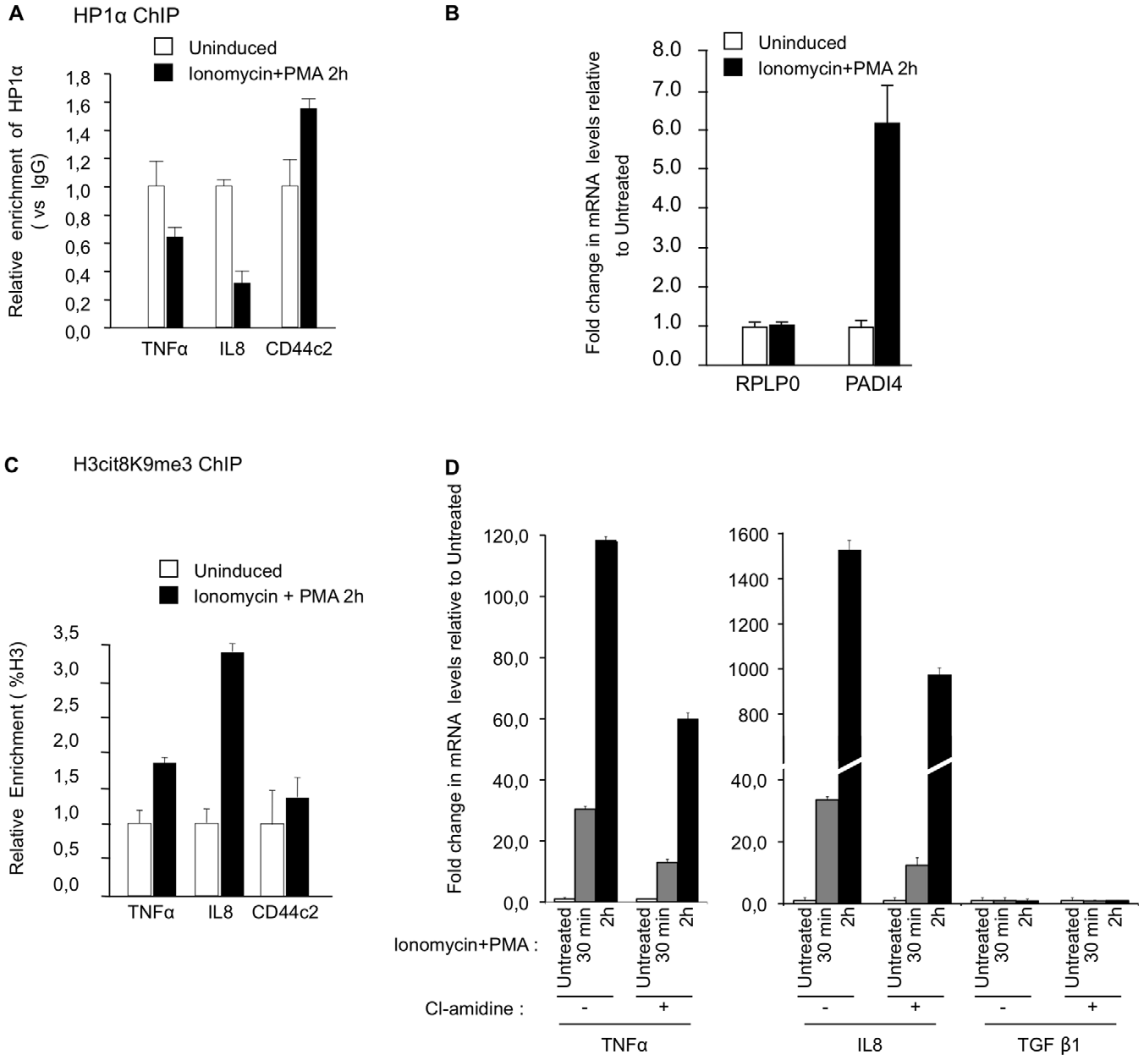
PADI activity facilitates activation of TNFα and IL8 transcription in T cells. (A) ChIP with anti-HP1α antibodies was carried out with chromatin from Jurkat cells either untreated or treated with PMA and ionophore (ionomycin). The relative enrichments of HP1α on the indicated promoters were measured by qPCR. Data are presented relative to non-immune IgG. Changes in enrichment are presented relative to the un-induced control (set to 1). Values are means ± SEM from two PCR measures of two independent ChIP experiments. (B) Total RNA was isolated from Jurkat cells either un-stimulated or treated with ionomycin and PMA for 2 hour. Changes in mRNA levels for the indicated genes were quantified by RT-qPCR. The data are presented as the means ± SEM of triplicate experiments. (C) ChIP with anti-H3cit8K9me3 antibodies was carried out as in A. Data are presented as a percentage of histone H3. Changes in enrichment are presented relative to the un-induced control (set to 1). Values are means ± SEM from two PCR measures of two independent ChIP experiments. (D) Total RNA was isolated from Jurkat cells treated as in B minus or plus PADI-inhibitor cl-amidine as indicated. Changes in mRNA levels for the indicated genes were quantified by RT-qPCR. The data are presented as the means ± SEM of duplicate experiments.

An inflammatory response can be induced in Jurkat T cells stimulated with an ionophore and the phorbol ester PMA. The stimulation of the Jurkat cells correlates with an eviction of HP1α from the promoter region of TNFα and IL8 (Figure 5A), and also from HERV-H/LTR62 and HERV-W/LTR (Figure S3A). The treatment also results in an approx. 6-fold increase of PADI4 accumulation (Figure 5B) and is expected to increase PADI activity as a consequence of the ionophore-induced calcium influx. We therefore used this system to determine whether increased PADI activity is associated with normal transcriptional activation of immune genes. Stimulation of the Jurkat cells resulted in a rapid and very transient recruitment of PADI4 to the promoters of *TNFα* and *IL8*, and at HERV-H/LTR62 and HERV-W/LTR (Figure S3B). This recruitment correlated with increase levels of H3Cit8K9me3 at these positions (Figure 5C and Figure S3C). Finally, inhibition of PADI activity with Cl-amidine reduced the kinetic and the abundance of *TNFα* and *IL8* mRNA accumulation in Jurkat cells stimulated by ionomycin and PMA (Figure 5D and ). Together, these observations suggest that PADI activity participates in the modification of the epigenetic landscape at the promoter of immune genes upon stimulation of T cells, and thereby facilitate the transcriptional activation of these genes.

### Reduced recruitment of HP1α on the promoter of the TNFα gene and on several HERVs in MS patients

**Figure 6:**
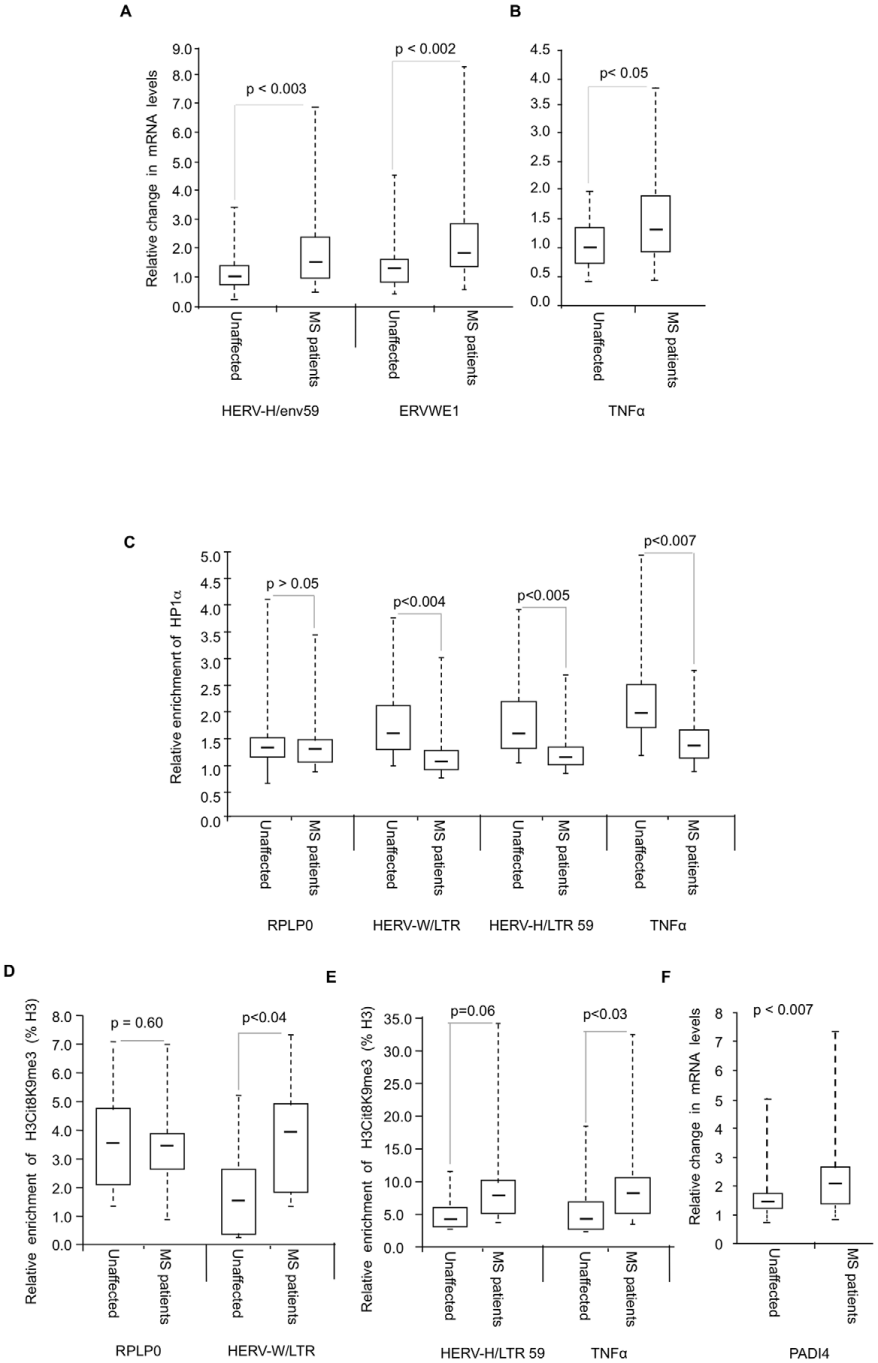
Modified accumulation of HP1α and H3cit8K9me3 at HERVs and TNFα in MS patients. (A–B) Expression of HERVs and TNFα in PBMCs from 18 families (n = 36 individuals) each consisting of one MS patient and a genetically related unaffected relative. Total RNA was prepared from the PBMCs and indicated transcripts were quantified by RT-qPCR. Values are means ±SE from at least 3 qPCR reactions for each individual. For each family, the basal level used to calculate the fold-change was the average of at least 3 qPCR values obtained from the healthy control. (C) ChIP with anti-HP1α on chromatin extracted at same time from PBMCs from above mentioned 18 families. The relative enrichment of HP1α on the LTR or promoter region of HERVs and TNFα was measured by qPCR and normalized to the signal obtained with irrelevant non-immune IgGs. The reported values are means ± SEM from at least three qPCRs for each individual (n = 36) from a representative ChIP experiment (* p<0.05, significant and °p>0.05 not significant, Wilcoxon signed rank test). (D–E) ChIP with anti-H3cit8K9me3 on chromatin extracted from PBMCs from 9 families. The relative enrichment of H3cit8K9me3 on the LTR or promoter region was measured by qPCR and data are presented as a percentage of histone H3. The reported values are means ± SEM from at least three qPCRs for each individual (n = 18) from a representative ChIP experiment. The enrichment of H3cit8K9me3 on the HERV/LTR59 is also higher in MS patients as compared to healthy individuals, but due to the small sample size, the p value (*° p = 0.06) is not significant and could not be interpreted (* p<0.05, significant and °p>0.05, not significant, Wilcoxon signed rank test). (F) PADI4 mRNA levels were quantified by RT-qPCR in total RNA prepared from PBMCs of 18 families each including one MS patients and one genetically related control individual. Values are means +/−SE from at least 3 qPCR reactions for each individual. The p value (p<0.0007) was calculated by Wilcoxon signed rank test. In each panel, the boxes represent the interquartile range. The whiskers extend the box to the highest and lowest value. The line across the box indicates the median value.

To investigate whether levels of HP1α recruitment and H3cit8K9me3 double modification at HERV and cytokine promoters were affected in MS, we collected PBMCs from 18 families, each family consisting of one MS patient and a genetically related healthy control (Table S1). The patients suffered from either relapsing-remitting (n = 10), or secondary progressive (n = 8) MS. As PBMCs yield only minute amounts of chromatin, our analysis was restricted to the LTRs of the unique HERV-H locus LTR59 [67] and the unique HERV-W locus ERVWE1 [68], and to the promoter of the cytokine TNFα. Consistent with previous observations [4], [5], [6], [7], transcription of these loci was significantly augmented in the MS patients (Figure 6A–6B).

ChIP assays revealed that recruitment of HP1α to the *TNFα* and the examined HERV promoters was significantly reduced in the MS patients compared to their genetically related healthy controls, while recruitment to a control promoter (RPLP0) was unchanged (Figure 6C). We also observed a significant correlation between HP1α levels on the *TNFα* promoter and HP1α levels on the LTRs of the HERVs, further suggesting that a single pathway regulates HP1α binding to both types of transcription units (Figure S4).

In ChIP assays, the anti-H3cit8K9me3 antibody was functional, but with a relatively poor sensitivity. Therefore, our analysis was restricted to the 9 families (9 patients and their respective healthy relatives) from whom we had the most abundant material. In these samples, levels of H3cit8K9me3 at the promoters of HERV-W/ERVWE1 and *TNFα* were significantly increased in the patients when compared to the genetically related healthy controls (Figure 6D–6E). On HERV-H/LTR59, levels of H3cit8K9me3 also appeared increased in the patients, but the p value associated with this data (0.06) is above the significance level of 0.05.

We finally questioned whether PADI4 could be linked to the increased levels of H3cit8K9me3. To this end, we examined PBMCs collected from the 18 families described above. Analysis of these samples by RT-PCR showed that PADI4 mRNA levels were significantly elevated in MS patients compared to the genetically related healthy controls (approx. 1.5-fold, Figure 6F).

Altogether, these experiments showed that in the patients, increased expression HERV-W/ERVWE1 and *TNFα* transcripts and decreased recruitment of HP1α at their promoter region is accompanied by a local increase in H3R8 citrullination and a moderate up-regulation of PADI4 expression.

## Discussion

In this report, we show that dependence on HP1α-mediated silencing is a common denominator between cytokines and HERVs, both expressed at abnormally high levels in T cells from MS patients, and we suggest that a decreased efficiency of the HP1-mediated silencing may participate in the pathological deregulation of these transcription units.

In this context, we find that one source of defective HP1-mediated silencing is citrullination of H3R8. This histone modification reduces the affinity of the chromo domain of the HP1 proteins to the methylated histone H3K9 residue and thereby defines a novel mechanism regulating HP1-binding to chromatin.

Using an antibody specifically recognizing H3cit8K9me3, we show that this double modification is induced in the presence of elevated levels of PADI4, the only known nuclear peptidylarginine deiminase. Interestingly, when an inflammatory response is induced in Jurkat T cells, expression of PADI4 is increased and levels of H3cit8K9me3 rise at the promoters of the immune genes *IL8* and *TNFα*. Under these conditions, inhibiting PADI activity with the chemical inhibitor Cl-amidine results in reduced kinetic and amplitude in the activation of the two immune genes. These observations show that PADI activity and citrullination of histone H3 are required for normal activation of immune genes and define PADI4 as a novel regulator of cytokine expression. We speculate that H3cit8, together with other histone modifications such as H3S10 and H3S28 phosphorylation participate in creating an epigenetic landscape favorable for the transcriptional activation of a subset of immune genes.

**Figure 7:**
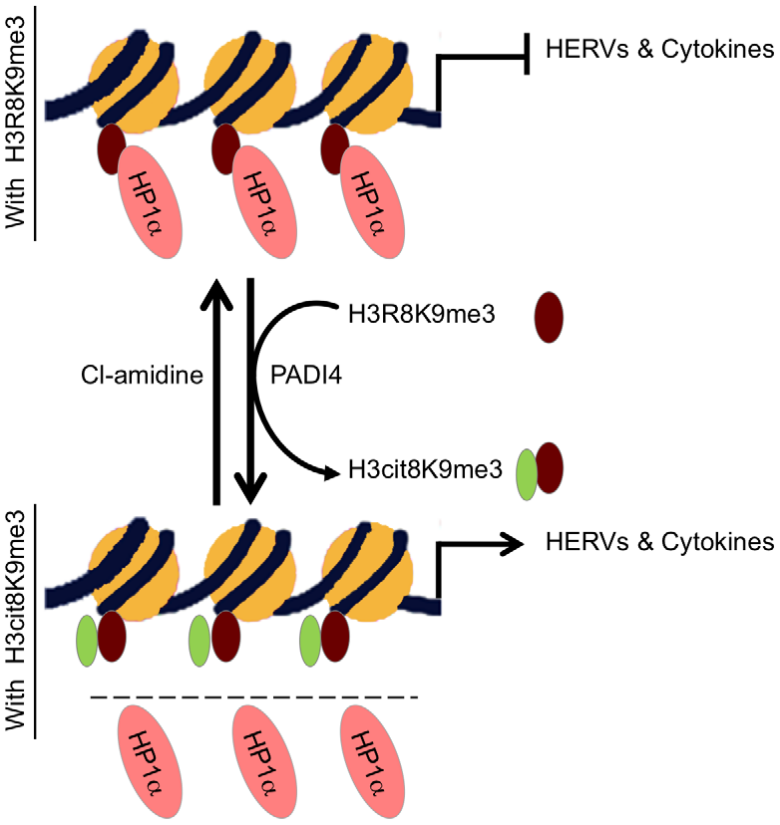
Model. HP1α silences HERVs and represses several cytokines until they are stimulated. It binds the tri-methylated lysine 9 of histone H3 on these promoters.This binding is in part regulated by PADI4 conversion of histone H3 arginine 8 into a citrulline, thereby reducing the affinity of HP1α for the neighboring methylated lysine 9. In MS patients, PADI4 induces an enrichment of H3cit8K9me3 double histone modification resulting in reduced accumulation of HP1α on HERVs, on the promoter of TNFα and possibly on other cytokines genes. This in turn may be responsible for excessive expression of HERVs and cytokines that inappropriately activate T lymphocytes and ultimately damages the central nervous system. It may be possible to interrupt this sequence of event with the PADI4 specific inhibitor Cl-amidine.

PADI4 activity could also be artificially increased in MCF7 cells treated with estradiol and an ionophore. This allowed us to show that abnormally elevated levels of PADI activity result in transcriptional stimulation of several immune genes. Consistent with this, PBMCs collected from MS patients (and compared to healthy relatives) showed in average increased expression of *TNFα*, increased levels of H3cit8K9me3 at the promoter of this gene, and increased expression of PADI4. These observations strongly suggest that inappropriate activity of PADI4 can participate in the deregulation of immune genes relevant for MS (see model Figure 7). We here note that the estrogen/ionophore treatment inducing PADI4 expression in MCF7 cells also stimulated production of this enzyme in Jurkat T cells (data not shown). We therefore speculate that PADI4 could be involved in the activating effect of estrogen on TNFα expression observed in T cells under some conditions ([69] and references therein) and could thereby play a role in the higher incidence of MS in females [70].

Other enzymes affecting the affinity of HP1 proteins for chromatin may also be good candidates for an implication in MS. For example, Jak2 that is expressed at increased levels in MS Th17 cells [71] also cause exclusion of HP1α from chromatin by phosphorylating H3Y41, a residue contacted by the C-terminal region of the HP1 proteins [72]. Along the same lines, we note that levels of arginine methylation of myelin basic protein MBP is increased in MS patients [73], while we find that methylation of H3R8 reduces affinity of HP1α for the neighboring methylated H3K9 approximately 10-fold. Possibly, the same arginine methylases may be involved in the modification of both MBP and histones. In addition, PADI4 has earlier been described as involved in arginine demethylation, although methylated arginines are rather poor substrates for this enzyme *in vitro* [44], [45], [47]. Methylation and citrullination may therefore allow for a gradual activation of HP1α target genes in response to external stimuli.

The fact that PADI4 is a regulator of cytokines that can be either positively regulated by cellular stimuli or negatively regulated by specific inhibitors provides yet unexplored avenues to the control of inflammation, and in the case of MS, molecules such as Cl-amidine may potentially allow restoring chromatin-mediated repression of over-activated cytokine genes.

While HP1 proteins are best described as heterochromatic silencers and suppressors of variegation, our observations confirm that these proteins are also highly relevant for the transcriptional control of inducible genes that require a transient phase of silencing. The sharing of regulatory mechanisms between euchromatic cytokine genes and repeated sequences such as HERVs suggests that many bridges may exist between active and inactive chromatin, and that there is a continuum and not a clear-cut boarder between euchromatin and heterochromatin. Therefore, probing the status of heterochromatic silencing as well as its defects may provide much new insight on the transcriptional programs in which cells are engaged.

## Materials and Methods

### Study population: Ethics statement

Blood samples were collected from each participant after informed consent as approved by the local Danish ethical committee.

### Study population: Description and methods

The study population consisted of 36 subjects, encompassing 18 MS patients clinically diagnosed for MS and fulfilling the diagnostic criteria of Poser et al., 1993 [75] and 18 unaffected (healthy) first or second degree relatives, one for each of the MS patients. The participants were from a homogenous population (Caucasian, Northern European descent). Venous blood was drawn and processed on the same day in our laboratory. The clinical and demographic data of each participant are summarized in Table S1. The mean age of both the MS patients and their unaffected relatives was 52 years. The gender ratios for MS patients (11 female/7 male) and unaffected relatives (9 female/9 male) were also comparable. Peripheral blood mononuclear cells (PBMCs) were prepared by standard Isopaque-Ficoll centrifugation. The separated cells were cryo-preserved in RPMI with addition of 20% inactivated human serum (HS) and 10% DMSO, at −135°C until use. For the assays, PBMCs were thawed and cultured for 24 h in RPMI-1640 with 10% inactivated human serum, and 100 Uml-1 penicillin-streptomycin at 37°C in a 5% CO_2_ incubator prior to use. For each family, PBMCs from the patient and the control individual were analyzed at the same time.The data were analyzed by using the software XLSTAT (version 2010.5.06, www.xlstat.com). When indicated in the text, Wilcoxon signed rank test [76] was used to determine whether a significant (p<0.05) difference.

### Antibodies, peptides, and chemicals

Anti-H3cit8K9me3 antibody was produced in rabbits using a peptide coupled to KLH with the following sequence: ARTKQTA (cit)(Kme3)STGGKAPRC. Anti-PADI4 (ChIP: P4749; immunoblots: ab50332), anti-H3cit(2,8,17) (ab5103), anti-H3(ab1791), and anti-H3K9me3 (ab8898) antibodies were purchased from Abcam. Anti-HP1α (ChIP: 1H5; immunoblots: 2G9) and anti-Brg1 (2E12) were from Euromedex. Calcium ionophore A23187 (used on HEK293 and MCF7 cells) and ionomycin (only ionophore tolerated by Jurkat cells), and 17-ß-estradiol (E2758) were purchased from Sigma. DNA was labeled with 4′,6-diamidino-2-phenylindole (DAPI; Invitrogen) at a concentration of 150 ng.ml^−1^. The peptide ARTKQTARKSTGGKAPRC was used for competition, overlay, and surface plasmon experiments, either unmodified or with K9me3, R8meK9me3, cit8K9me3, or cit8 modifications. Peptides were carefully quantified by amino acid analysis, and the presence of the modifications was confirmed by mass spectrometry.

### Chromatin immunoprecipitation (ChIP)

ChIP was carried out essentially as previously described [74], with minor alterations. MCF7 or Jurkat cells, or PBMCs were cross-linked in phosphate-buffered saline (PBS) containing 1% formaldehyde (Sigma) for 10 min at room temperature. The crosslinking reaction was quenched with PBS containing 125 mM glycine, followed by three washes with ice-cold PBS. The chromatin was fragmented by sonication to produce average DNA lengths of 0.5 kb. After ChIP, the eluted DNAs were detected by quantitative PCR using the primers listed in Table S2. Levels of histone modifications are expressed as % of H3, and levels of HP1α are expressed relatively to the signal obtained for ChIP using non-immune IgGs. Values are averaged from 3 independent experiments.

### *In vitro* binding studies by surface plasmon resonance (SPR)

Real-time SPR assays were performed at 25°C in PBS. GST-HP1α was covalently coupled to a CM5 sensor chip, using a Biacore 2000 instrument and an Amine Coupling Kit (GE Healthcare), achieving three different immobilization densities (R_immo_) of 3500, 6500, and 15000 resonance units (RU; 1RU ≈1 pg.mm^−2^). On the remaining flow cell, 5700 RU of GST were immobilized to prepare a reference surface. A series of 10 concentrations of peptides (50 nM–25 µM for H3R8K9me3, 200 nM–100 µM for the H3cit8K9me3, H3cit8K9, and unmodified peptides) were injected for 2 min over the GST-HP1α and GST surfaces at a flow rate of 50 µL.min^−1^. After following the dissociation for 5 min, the surfaces were regenerated with a 3-min wash of 2 M NaCl, and two 15-sec washes with 10 mM glycine-HCl (pH 1.5) and 0.05% SDS.

The association and dissociation profiles were double-referenced using the Scrubber 2.0 software (BioLogic Software) (*i.e.* both the signals from the reference GST surface and from blank experiments using PBS instead of peptide were subtracted). The steady-state SPR responses (R_eq_) were plotted against the peptide concentration (C) and fitted according to the following equation:
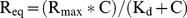
(1)where K_d_ is the equilibrium dissociation constant of the peptide/GST-HP1α interaction and R_max_ the maximal binding capacity of GST-HP1α.

The percentage of bound HP1α sites was determined as follows:
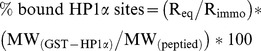
(2)


### Cell culture

MCF-7 and HEK293 cells were cultured in Dulbecco's modified Eagle's medium (DMEM, Gibco BRL), and Jurkat cells were cultured in RPMI-1640, all with 10% decomplemented fetal bovine serum (FBS) and 100 U.ml^−1^ penicillin-streptomycin at 37°C in a 5% CO_2_ incubator. MCF7 cells were treated with 200 nM of estradiol (E2) for 24 h, washed three times with 1× PBS and then incubated for 30 minutes in Locke's solution (10 mM HEPES.HCl, pH 7.3, 150 mM NaCl, 5 mM KCl, 2 mM CaCl_2_, and 0.1% glucose) supplemented with 5 µM A23187 (C7522, Sigma). Jurkat cells were treated with phorbol myristate acetate (PMA) at 40 nM. Ionophores were used at 1 µM. PADI4 inhibitor, Cl-amidine (from Bertin Pharma) was dissolved in 1× PBS as a 50 mM stock solution. MCF7 and Jurkat cells were treated with 200 µM Cl-amidine in complete cell culture medium for exactly 72 h.

### Generation of inducible PADI4-expressing HEK293 cells

The cDNAs coding for either WT PADI4, hyperactive PADI4 (Mut+) [62], or hypoactive PADI4 (Mut−) [63] were inserted into the pi_tk_hygro vector for retroviral delivery [77]. Two days after transfection using FuGENE (Roche), the medium of packaging HEK293 cells was filtered on a 0.45 µm filter (Millipore) and supplemented with polybrene (AL-118, Sigma) at 100 µg.ml^−1^. This medium was used to infect host HEK293 cells (3 consecutive infections) followed by selection with hygromycin (H3274, Sigma) at a final concentration of 100 µg.ml^−1^.

### RNA interference, mRNA, and protein quantification

HP1 small interfering RNAs (siRNAs) were described previously [35]. PADI4 (J-012471-05) and Glyceraldehyde-3-phosphate dehydrogenase control siRNAs were purchased from Dharmacon. Cells were harvested 72 h after transfection with DharmaFECT 1 (T 2001-03). Total RNA from PBMCs and MCF7 cells was extracted with RNeasy (Qiagen) and quantified with an ND-1000 (Nanodrop). After DNase treatment (Roche), reverse transcription was performed using SuperScript III (Invitrogen) and random hexanucleotides according to the manufacturer's instructions. Complementary DNA was quantified by RT-qPCR as previously described [35]. PCR primers are listed in Table S2. Proteins were extracted as described previously [35] and detected by Western blotting. Acid extraction of histones was conducted as described by Shechter, *et al.,* [78].

### Immunofluorescence and image acquisition

Immunofluorescent labeling was performed in MCF7 cells after sequential treatment with 200 nM of estradiol (E2) for 24 h in culture media followed by 5 µM A23187 for 30 min in Locke's solution. Cells were permeabilized in ice cold CSK (20 mM PIPES pH 6.8, 200 mM NaCl, 600 mM sucrose, 6 mM MgCl_2_, and 2 mM EGTA) 0.5% Triton X-100 (v/v), 0.1 mM PMSF for 30 sec. Cells were fixed with CSK-3.7% paraformaldehyde at room temperature (RT) for 10 min, then blocked in PBS-0.05% Tween-20 (v/v), 10% (v/v) FBS for 30 min at RT. In peptide competition experiments, primary antibodies were pre-incubated with 1 µg of indicated histone H3 peptides or without peptide for 30 min. Cells were incubated with antibodies at 4°C overnight and then coverslips were washed three times in PBS-BSA 0.5% (w/v). Coverslips were incubated with secondary antibodies for 1 h at room temperature protected from light, before being washed three times in PBS-BSA 0.5% (w/v), and once in PBS, before final staining with DAPI. Imaging was conducted on an Axiovert 200 M microscope (Zeiss) coupled with an Apotome with Axiovision 4.7 (Zeiss).

## Supporting Information

Figure S1 Depletion of HP1 proteins reactivates HERVs and TNFα. (A) Total RNA from MCF7 cells transfected with the indicated small interfering RNAs (siRNAs) was quantified with RT-qPCR. Changes in mRNA levels are shown relative to the siGAPDH transfection (set to 1). The data are presented as the means ± SEM of triplicate experiments.(B and D) MCF7 cells were transfected with the indicated siRNA as mentioned in Figure 1B and Figure 3E–3F. Western blots were carried out with total extracts with the indicated antibodies. Blots are representative of the experimental replicates.
(PDF)

Figure S2 PADI4 expression and activity is induced in MCF7 cells by a treatment with estradiol and an ionophore. (A) Extracts from MCF7 cells treated either estradiol (E2) or ionophore (A23187) and/or both as indicated were analyzed by Western-blot with anti-PADI4 & anti-Brg1 antibodies. Blots are representative of the experimental replicates. (B) Treatment of MCF7 cells with estradiol and an ionophore increases the expression of HERV-H/env62 in 24 h. Total RNA from MCF7 cells uninduced (ethanol) or treated with E2 and/or A23187 for the indicated times, was quantified by RT-qPCR. Values were normalized to levels of RPLP0. Indicated values were averaged from three experimental replicates. (C) PADI4 activity does not reduce recruitment of H3cit8K9me3 on the promoter of *RPLP0* in MCF7 cells. Relative enrichment of H3cit8K9me3 double mark on the *RPLP0* promoter under the different condition as described in Figure 4.
(PDF)

Figure S3 PADI activity facilitates activation of HERVs in T cells. (A) ChIP with anti-HP1α antibodies was carried out with chromatin prepared from Jurkat cells either untreated or treated with PMA and ionophore (ionomycin). The relative enrichments of HP1α on the indicated LTRs were measured by qPCR. Data are presented relative to non-immune IgG. Changes in enrichment are presented relative to the un-induced control (set to 1). Values are means ± SEM from two PCR measures of two independent ChIP experiments. (B) ChIP with anti-PADI4 antibodies was carried out as in A with indicated time points. Data are presented relative to non-immune IgG. Changes in enrichment are presented relative to the un-induced control (set to 1). Values are means ± SEM from two PCR measures of two independent ChIP experiments. (C) ChIP with anti-H3cit8K9me3 antibodies was carried out as in A. Data are presented as a percentage of histone H3. Changes in enrichment are presented relative to the un-induced control (set to 1). Values are means ± SEM from two PCR measures of two independent ChIP experiments. (D) Total RNA was isolated from Jurkat cells either un-stimulated or treated with ionomycin and PMA minus or plus PADI-inhibitor cl-amidine as indicated. Changes in mRNA levels for the indicated genes were quantified by RT-qPCR. The data are presented as the means ± SEM of duplicate experiments.
(PDF)

Figure S4 HP1α in the repression of HERVs and cytokines. Correlation matrix representing the correlation between the presence of HP1α on the promoter regions of RPLP0, TNFα, and HERVs in study individuals analyzed in Figure 6C. Represented values are Spearman Rank correlation coefficients followed by p-values calculated using two sided student t test. p<0.05 was considered as significant. The colour bar at the bottom shows the colour scale according to Spearman's Rank correlation coefficients.
(PDF)

Table S1 Clinical and demographic data for the lymphocyte samples. The data given for age and duration (years), and EDSS are: mean (range). Abbreviations are as follows: MS: MS patient; U: unaffected relative; PP primary progressive MS; RR: relapsing-remitting MS; SP: secondary progressive MS; M: male; F: female; y: years; EDSS: expanded disability status scale; active: disease course with relapse within a year prior to sampling. None of the patients had diagnosed infections at the time of sampling.
(DOC)

Table S2 List of primers used in this study.
(DOC)

## References

[1] Noseworthy JH, Lucchinetti C, Rodriguez M, Weinshenker BG (2000) Multiple sclerosis. N Engl J Med 343: 938–952. 11006371 10.1056/NEJM200009283431307

[2] O'Brien K, Gran B, Rostami A (2010) T-cell based immunotherapy in experimental autoimmune encephalomyelitis and multiple sclerosis. Immunotherapy 2: 99–115. 20231863 10.2217/imt.09.61 PMC2837464

[3] Chitnis T (2007) The role of CD4 T cells in the pathogenesis of multiple sclerosis. International review of neurobiology 79: 43–72. 17531837 10.1016/S0074-7742(07)79003-7 PMC7112308

[4] Imitola J, Chitnis T, Khoury SJ (2005) Cytokines in multiple sclerosis: from bench to bedside. Pharmacol Ther 106: 163–177. 15866318 10.1016/j.pharmthera.2004.11.007

[5] Brudek T, Christensen T, Aagaard L, Petersen T, Hansen HJ, et al (2009) B cells and monocytes from patients with active multiple sclerosis exhibit increased surface expression of both HERV-H Env and HERV-W Env, accompanied by increased seroreactivity. Retrovirology 6: 104. 19917105 10.1186/1742-4690-6-104 PMC2780989

[6] Christensen T (2010) HERVs in neuropathogenesis. J Neuroimmune Pharmacol 5: 326–335. 20422298 10.1007/s11481-010-9214-y

[7] Perron H, Lang A (2010) The human endogenous retrovirus link between genes and environment in multiple sclerosis and in multifactorial diseases associating neuroinflammation. Clin Rev Allergy Immunol 39: 51–61. 19697163 10.1007/s12016-009-8170-x

[8] Deaton AM, Bird A (2011) CpG islands and the regulation of transcription. Genes & development 25: 1010–1022. 21576262 10.1101/gad.2037511 PMC3093116

[9] Walsh CP, Chaillet JR, Bestor TH (1998) Transcription of IAP endogenous retroviruses is constrained by cytosine methylation. Nature Genetics 20: 116–117. 9771701 10.1038/2413

[10] Mikkelsen TS, Ku M, Jaffe DB, Issac B, Lieberman E, et al (2007) Genome-wide maps of chromatin state in pluripotent and lineage-committed cells. Nature 448: 553–560. 17603471 10.1038/nature06008 PMC2921165

[11] Hutnick LK, Huang X, Loo T-C, Ma Z, Fan G (2010) Repression of retrotransposal elements in mouse embryonic stem cells is primarily mediated by a DNA methylation-independent mechanism. Journal of Biological Chemistry 285: 21082–21091. 20404320 10.1074/jbc.M110.125674 PMC2898347

[12] Matsui T, Leung D, Miyashita H, Maksakova IA, Miyachi H, et al (2010) Proviral silencing in embryonic stem cells requires the histone methyltransferase ESET. Nature 464: 927–931. 20164836 10.1038/nature08858

[13] Karimi MM, Goyal P, Maksakova IA, Bilenky M, Leung D, et al (2011) DNA methylation and SETDB1/H3K9me3 regulate predominantly distinct sets of genes, retroelements, and chimeric transcripts in mESCs. Cell stem cell 8: 676–687. 21624812 10.1016/j.stem.2011.04.004 PMC3857791

[14] Martens JHA, O'Sullivan RJ, Braunschweig U, Opravil S, Radolf M, et al (2005) The profile of repeat-associated histone lysine methylation states in the mouse epigenome. EMBO J 24: 800–812. 15678104 10.1038/sj.emboj.7600545 PMC549616

[15] Mulligan P, Westbrook TF, Ottinger M, Pavlova N, Chang B, et al (2008) CDYL bridges REST and histone methyltransferases for gene repression and suppression of cellular transformation. Molecular Cell 32: 718–726. 19061646 10.1016/j.molcel.2008.10.025 PMC6595072

[16] Fischle W, Franz H, Jacobs SA, Allis CD, Khorasanizadeh S (2008) Specificity of the chromodomain Y chromosome family of chromodomains for lysine-methylated ARK(S/T) motifs. The Journal of biological chemistry 283: 19626–19635. 18450745 10.1074/jbc.M802655200 PMC2443675

[17] Bernstein E, Duncan EM, Masui O, Gil J, Heard E, et al (2006) Mouse polycomb proteins bind differentially to methylated histone H3 and RNA and are enriched in facultative heterochromatin. Molecular and cellular biology 26: 2560–2569. 16537902 10.1128/MCB.26.7.2560-2569.2006 PMC1430336

[18] Jacobs SA, Taverna SD, Zhang Y, Briggs SD, Li J, et al (2001) Specificity of the HP1 chromo domain for the methylated N-terminus of histone H3. The EMBO Journal 20: 5232–5241. 11566886 10.1093/emboj/20.18.5232 PMC125272

[19] Bannister AJ, Zegerman P, Partridge JF, Miska EA, Thomas JO, et al (2001) Selective recognition of methylated lysine 9 on histone H3 by the HP1 chromo domain. Nature 410: 120–124. 11242054 10.1038/35065138

[20] Lachner M, O'Carroll D, Rea S, Mechtler K, et al (2001) Methylation of histone H3 lysine 9 creates a binding site for HP1 proteins. Nature 410: 116–120. 11242053 10.1038/35065132

[21] Kim J, Daniel J, Espejo A, Lake A, Krishna M, et al (2006) Tudor, MBT and chromo domains gauge the degree of lysine methylation. EMBO reports 7: 397–403. 16415788 10.1038/sj.embor.7400625 PMC1456902

[22] Iwase S, Lan F, Bayliss P, de la Torre-Ubieta L, Huarte M, et al (2007) The X-linked mental retardation gene SMCX/JARID1C defines a family of histone H3 lysine 4 demethylases. Cell 128: 1077–1088. 17320160 10.1016/j.cell.2007.02.017

[23] Rottach A, Frauer C, Pichler G, Bonapace IM, Spada F, et al (2010) The multi-domain protein Np95 connects DNA methylation and histone modification. Nucleic acids research 38: 1796–1804. 20026581 10.1093/nar/gkp1152 PMC2847221

[24] Wolf D, Hug K, Goff SP (2008) TRIM28 mediates primer binding site-targeted silencing of Lys1,2 tRNA-utilizing retroviruses in embryonic cells. Proceedings of the National Academy of Sciences of the United States of America 105: 12521–12526. 18713861 10.1073/pnas.0805540105 PMC2518094

[25] Rowe HM, Jakobsson J, Mesnard D, Rougemont J, Reynard S, et al (2010) KAP1 controls endogenous retroviruses in embryonic stem cells. Nature 463: 237–240. 20075919 10.1038/nature08674

[26] Maksakova IA, Goyal P, Bullwinkel J, Brown JP, Bilenky M, et al (2011) H3K9me3-binding proteins are dispensable for SETDB1/H3K9me3-dependent retroviral silencing. Epigenetics & chromatin 4: 12. 21774827 10.1186/1756-8935-4-12 PMC3169442

[27] Eissenberg JC, Elgin SC (2000) The HP1 protein family: getting a grip on chromatin. Curr Opin Genet Dev 10: 204–210. 10753776 10.1016/s0959-437x(00)00058-7

[28] Kwon SH, Workman JL (2008) The heterochromatin protein 1 (HP1) family: put away a bias toward HP1. Mol Cells 26: 217–227. 18664736

[29] El Gazzar M, Yoza BK, Hu JY, Cousart SL, McCall CE (2007) Epigenetic silencing of tumor necrosis factor alpha during endotoxin tolerance. J Biol Chem 282: 26857–26864. 17646159 10.1074/jbc.M704584200

[30] Yoza BK, McCall CE (2011) Facultative heterochromatin formation at the IL-1 beta promoter in LPS tolerance and sepsis. Cytokine 53: 145–152. 21078560 10.1016/j.cyto.2010.10.007 PMC3021647

[31] Chen X, El Gazzar M, Yoza BK, McCall CE (2009) The NF-kappaB factor RelB and histone H3 lysine methyltransferase G9a directly interact to generate epigenetic silencing in endotoxin tolerance. J Biol Chem 284: 27857–27865. 19690169 10.1074/jbc.M109.000950 PMC2788836

[32] Ndlovu MN, Van Lint C, Van Wesemael K, Callebert P, Chalbos D, et al (2009) Hyperactivated NF-{kappa}B and AP-1 transcription factors promote highly accessible chromatin and constitutive transcription across the interleukin-6 gene promoter in metastatic breast cancer cells. Mol Cell Biol 29: 5488–5504. 19687301 10.1128/MCB.01657-08 PMC2756884

[33] Saint-André V, Batsché E, Rachez C, Muchardt C (2011) Histone H3 lysine 9 trimethylation and HP1γ favor inclusion of alternative exons. Nat Struct Mol Biol 10.1038/nsmb.1995 21358630

[34] Lavigne M, Eskeland R, Azebi S, Saint-André V, Jang SM, et al (2009) Interaction of HP1 and Brg1/Brm with the Globular Domain of Histone H3 Is Required for HP1-Mediated Repression. PLoS Genet 5: e1000769 doi:10.1371/journal.pgen.1000769. 20011120 10.1371/journal.pgen.1000769 PMC2782133

[35] Mateescu B, Bourachot B, Rachez C, Ogryzko V, Muchardt C (2008) Regulation of an inducible promoter by an HP1beta-HP1gamma switch. EMBO Rep 9: 267–272. 18239689 10.1038/embor.2008.1 PMC2267390

[36] Marban C, Suzanne S, Dequiedt F, de Walque S, Redel L, et al (2007) Recruitment of chromatin-modifying enzymes by CTIP2 promotes HIV-1 transcriptional silencing. Embo J 26: 412–423. 17245431 10.1038/sj.emboj.7601516 PMC1783449

[37] Chéné I, Basyuk E, Lin YL, Triboulet R, Knezevich A, et al (2007) Suv39H1 and HP1gamma are responsible for chromatin-mediated HIV-1 transcriptional silencing and post-integration latency. Embo J 26: 424–435. 17245432 10.1038/sj.emboj.7601517 PMC1783455

[38] Pedersen MT, Helin K (2010) Histone demethylases in development and disease. Trends in Cell Biology 20: 662–671. 20863703 10.1016/j.tcb.2010.08.011

[39] Mateescu B, England P, Halgand F, Yaniv M, Muchardt C (2004) Tethering of HP1 proteins to chromatin is relieved by phosphoacetylation of histone H3. EMBO Rep 10.1038/sj.embor.7400139 PMC1299051 15105826

[40] Fischle W, Tseng BS, Dormann HL, Ueberheide BM, Garcia BA, et al (2005) Regulation of HP1-chromatin binding by histone H3 methylation and phosphorylation. Nature 438: 1116–1122. 16222246 10.1038/nature04219

[41] Vermeulen M, Eberl HC, Matarese F, Marks H, Denissov S, et al (2010) Quantitative Interaction Proteomics and Genome-wide Profiling of Epigenetic Histone Marks and Their Readers. Cell 142: 967–980. 20850016 10.1016/j.cell.2010.08.020

[42] Di Lorenzo A, Bedford MT (2011) Histone arginine methylation. FEBS LETTERS 585: 2024–2031. 21074527 10.1016/j.febslet.2010.11.010 PMC3409563

[43] Bedford MT, Clarke SG (2009) Protein arginine methylation in mammals: who, what, and why. Mol Cell 33: 1–13. 19150423 10.1016/j.molcel.2008.12.013 PMC3372459

[44] Cuthbert GL, Daujat S, Snowden AW, Erdjument-Bromage H, Hagiwara T, et al (2004) Histone deimination antagonizes arginine methylation. Cell 118: 545–553. 15339660 10.1016/j.cell.2004.08.020

[45] Wang Y, Wysocka J, Sayegh J, Lee YH, Perlin JR, et al (2004) Human PAD4 regulates histone arginine methylation levels via demethylimination. Science 306: 279–283. 15345777 10.1126/science.1101400

[46] Nakashima K, Hagiwara T, Yamada M (2002) Nuclear localization of peptidylarginine deiminase V and histone deimination in granulocytes. J Biol Chem 277: 49562–49568. 12393868 10.1074/jbc.M208795200

[47] Raijmakers R, Zendman AJ, Egberts WV, Vossenaar ER, Raats J, et al (2007) Methylation of arginine residues interferes with citrullination by peptidylarginine deiminases in vitro. J Mol Biol 367: 1118–1129. 17303166 10.1016/j.jmb.2007.01.054

[48] Migliori V, Phalke S, Bezzi M, Guccione E (2010) Arginine/lysine-methyl/methyl switches: biochemical role of histone arginine methylation in transcriptional regulation. Epigenomics 2: 119–137. 22122749 10.2217/epi.09.39

[49] Hagiwara T, Hidaka Y, Yamada M (2005) Deimination of histone H2A and H4 at arginine 3 in HL-60 granulocytes. Biochemistry 44: 5827–5834. 15823041 10.1021/bi047505c

[50] Li P, Yao H, Zhang Z, Li M, Luo Y, et al (2008) Regulation of p53 target gene expression by peptidylarginine deiminase 4. Mol Cell Biol 28: 4745–4758. 18505818 10.1128/MCB.01747-07 PMC2493360

[51] Yao H, Li P, Venters BJ, Zheng S, Thompson PR, et al (2008) Histone Arg modifications and p53 regulate the expression of OKL38, a mediator of apoptosis. J Biol Chem 283: 20060–20068. 18499678 10.1074/jbc.M802940200 PMC2459274

[52] Denis H, Deplus R, Putmans P, Yamada M, Metivier R, et al (2009) Functional connection between deimination and deacetylation of histones. Mol Cell Biol 29: 4982–4993. 19581286 10.1128/MCB.00285-09 PMC2738279

[53] Zhang X, Gamble MJ, Stadler S, Cherrington BD, Causey CP, et al (2011) Genome-Wide Analysis Reveals PADI4 Cooperates with Elk-1 to Activate c-Fos Expression in Breast Cancer Cells. PLoS Genet 7: e1002112 doi:10.1371/journal.pgen.1002112. 21655091 10.1371/journal.pgen.1002112 PMC3107201

[54] Suzuki A, Yamada R, Chang X, Tokuhiro S, Sawada T, et al (2003) Functional haplotypes of PADI4, encoding citrullinating enzyme peptidylarginine deiminase 4, are associated with rheumatoid arthritis. Nat Genet 34: 395–402. 12833157 10.1038/ng1206

[55] Ikari K, Kuwahara M, Nakamura T, Momohara S, Hara M, et al (2005) Association between PADI4 and rheumatoid arthritis: a replication study. Arthritis Rheum 52: 3054–3057. 16200584 10.1002/art.21309

[56] Kang CP, Lee HS, Ju H, Cho H, Kang C, et al (2006) A functional haplotype of the PADI4 gene associated with increased rheumatoid arthritis susceptibility in Koreans. Arthritis Rheum 54: 90–96. 16385500 10.1002/art.21536

[57] Anzilotti C, Pratesi F, Tommasi C, Migliorini P (2009) Peptidylarginine deiminase 4 and citrullination in health and disease. Autoimmun Rev 10.1016/j.autrev.2009.06.002 19540364

[58] Mastronardi FG, Wood DD, Mei J, Raijmakers R, Tseveleki V, et al (2006) Increased citrullination of histone H3 in multiple sclerosis brain and animal models of demyelination: a role for tumor necrosis factor-induced peptidylarginine deiminase 4 translocation. J Neurosci 26: 11387–11396. 17079667 10.1523/JNEUROSCI.3349-06.2006 PMC6674531

[59] Wang-Johanning F, Rycaj K, Plummer JB, Li M, Yin B, et al (2012) Immunotherapeutic potential of anti-human endogenous retrovirus-k envelope protein antibodies in targeting breast tumors. Journal of the National Cancer Institute 104: 189–210. 22247020 10.1093/jnci/djr540 PMC3274512

[60] Skundric DS, Cai J, Cruikshank WW, Gveric D (2006) Production of IL-16 correlates with CD4+ Th1 inflammation and phosphorylation of axonal cytoskeleton in multiple sclerosis lesions. J Neuroinflammation 3: 13. 16729885 10.1186/1742-2094-3-13 PMC1488832

[61] Vaknin-Dembinsky A, Balashov K, Weiner HL (2006) IL-23 is increased in dendritic cells in multiple sclerosis and down-regulation of IL-23 by antisense oligos increases dendritic cell IL-10 production. J Immunol 176: 7768–7774. 16751425 10.4049/jimmunol.176.12.7768

[62] Hung HC, Lin CY, Liao YF, Hsu PC, Tsay GJ, et al (2007) The functional haplotype of peptidylarginine deiminase IV (S55G, A82V and A112G) associated with susceptibility to rheumatoid arthritis dominates apoptosis of acute T leukemia Jurkat cells. Apoptosis 12: 475–487. 17216583 10.1007/s10495-006-0005-0

[63] Arita K, Shimizu T, Hashimoto H, Hidaka Y, Yamada M, et al (2006) Structural basis for histone N-terminal recognition by human peptidylarginine deiminase 4. Proc Natl Acad Sci U S A 103: 5291–5296. 16567635 10.1073/pnas.0509639103 PMC1459348

[64] Muchardt C, Guilleme M, Seeler JS, Trouche D, Dejean A, et al (2002) Coordinated methyl and RNA binding is required for heterochromatin localization of mammalian HP1alpha. EMBO Rep 3: 975–981. 12231507 10.1093/embo-reports/kvf194 PMC1307621

[65] Dong S, Zhang Z, Takahara H (2007) Estrogen-enhanced peptidylarginine deiminase type IV gene (PADI4) expression in MCF-7 cells is mediated by estrogen receptor-alpha-promoted transfactors activator protein-1, nuclear factor-Y, and Sp1. Mol Endocrinol 21: 1617–1629. 17456793 10.1210/me.2006-0550

[66] Luo Y, Arita K, Bhatia M, Knuckley B, Lee YH, et al (2006) Inhibitors and inactivators of protein arginine deiminase 4: functional and structural characterization. Biochemistry 45: 11727–11736. 17002273 10.1021/bi061180d PMC1808342

[67] de Parseval N, Casella J, Gressin L, Heidmann T (2001) Characterization of the three HERV-H proviruses with an open envelope reading frame encompassing the immunosuppressive domain and evolutionary history in primates. Virology 279: 558–569. 11162811 10.1006/viro.2000.0737

[68] Mallet F, Bouton O, Prudhomme S, Cheynet V, Oriol G, et al (2004) The endogenous retroviral locus ERVWE1 is a bona fide gene involved in hominoid placental physiology. Proc Natl Acad Sci USA 101: 1731–1736. 14757826 10.1073/pnas.0305763101 PMC341840

[69] Straub RH (2007) The complex role of estrogens in inflammation. Endocrine reviews 28: 521–574. 17640948 10.1210/er.2007-0001

[70] Orton SM, Herrera BM, Yee IM, Valdar W, Ramagopalan SV, et al (2006) Sex ratio of multiple sclerosis in Canada: a longitudinal study. The Lancet Neurology 5: 932–936. 17052660 10.1016/S1474-4422(06)70581-6

[71] Conti L, De Palma R, Rolla S, Boselli D, Rodolico G, et al (2012) Th17 Cells in Multiple Sclerosis Express Higher Levels of JAK2, Which Increases Their Surface Expression of IFN-γR2. Journal of immunology (Baltimore, Md: 1950) 188: 1011–1018. 10.4049/jimmunol.1004013 22219326

[72] Dawson MA, Bannister AJ, Gottgens B, Foster SD, Bartke T, et al (2009) JAK2 phosphorylates histone H3Y41 and excludes HP1alpha from chromatin. Nature 461: 819–822. 19783980 10.1038/nature08448 PMC3785147

[73] Kim JK, Mastronardi FG, Wood DD, Lubman DM, Zand R, et al (2003) Multiple sclerosis: an important role for post-translational modifications of myelin basic protein in pathogenesis. Molecular & cellular proteomics: MCP 2: 453–462. 12832457 10.1074/mcp.M200050-MCP200

[74] Batsché E, Yaniv M, Muchardt C (2006) The human SWI/SNF subunit Brm is a regulator of alternative splicing. Nat Struct Mol Biol 13: 22–29. 16341228 10.1038/nsmb1030

[75] Poser CM, Paty DW, Scheinberg L, McDonald WI, Davis FA, et al (1983) New diagnostic criteria for multiple sclerosis: guidelines for research protocols. Ann Neurol 13: 227–231. 6847134 10.1002/ana.410130302

[76] Wilcoxon F (1945) Individual comparisons by ranking methods. Biometrics Bulletin 1: 80–83.

[77] Stolarov J, Chang K, Reiner A, Rodgers L, Hannon GJ, et al (2001) Design of a retroviral-mediated ecdysone-inducible system and its application to the expression profiling of the PTEN tumor suppressor. Proc Natl Acad Sci U S A 98: 13043–13048. 11687610 10.1073/pnas.221450598 PMC60821

[78] Shechter D, Dormann HL, Allis CD, Hake SB (2007) Extraction, purification and analysis of histones. Nat Protoc 2: 1445–1457. 17545981 10.1038/nprot.2007.202

