# Supplementary figures and images for: Citrullination of Histone H3 Interferes with HP1-Mediated Transcriptional Repression
Source: PLoS Genet. 2012 Sep 13;8(9):e1002934. doi: 10.1371/journal.pgen.1002934 (PMC3441713; doi:10.1371/journal.pgen.1002934)

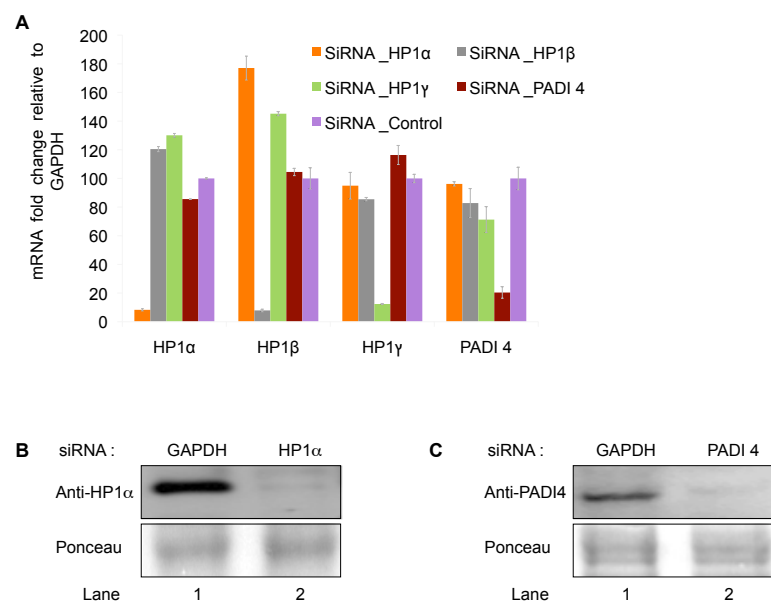

Figure S1 Sharma *et al.*

Supplement: Figure S1 — Depletion of HP1 proteins reactivates HERVs and TNFα. (A) Total RNA from MCF7 cells transfected with the indicated small interfering RNAs (siRNAs) was quantified with RT-qPCR. Changes in mRNA levels are shown relative to the siGAPDH transfection (set to 1). The data are presented as the means ± SEM of triplicate experiments.(B and D) MCF7 cells were transfected with the indicated siRNA as mentioned in Figure 1B and Figure 3E–3F. Western blots were carried out with total extracts with the indicated antibodies. Blots are representative of the experimental replicates. (PDF) [file pgen.1002934.s001.pdf]

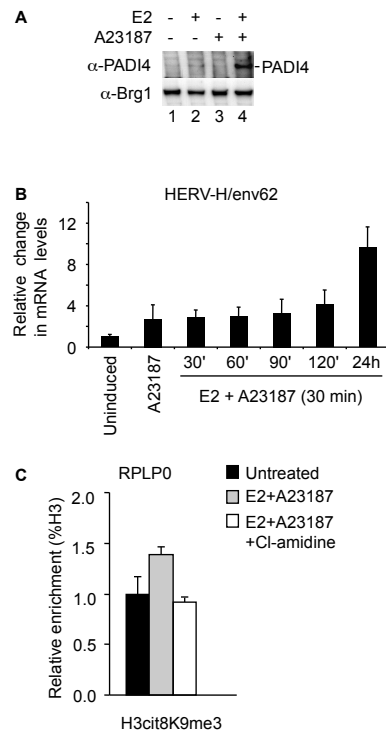

Figure S2 Sharma *et al.*

Supplement: Figure S2 — PADI4 expression and activity is induced in MCF7 cells by a treatment with estradiol and an ionophore. (A) Extracts from MCF7 cells treated either estradiol (E2) or ionophore (A23187) and/or both as indicated were analyzed by Western-blot with anti-PADI4 & anti-Brg1 antibodies. Blots are representative of the experimental replicates. (B) Treatment of MCF7 cells with estradiol and an ionophore increases the expression of HERV-H/env62 in 24 h. Total RNA from MCF7 cells uninduced (ethanol) or treated with E2 and/or A23187 for the indicated times, was quantified by RT-qPCR. Values were normalized to levels of RPLP0. Indicated values were averaged from three experimental replicates. (C) PADI4 activity does not reduce recruitment of H3cit8K9me3 on the promoter of RPLP0 in MCF7 cells. Relative enrichment of H3cit8K9me3 double mark on the RPLP0 promoter under the different condition as described in Figure 4. (PDF) [file pgen.1002934.s002.pdf]

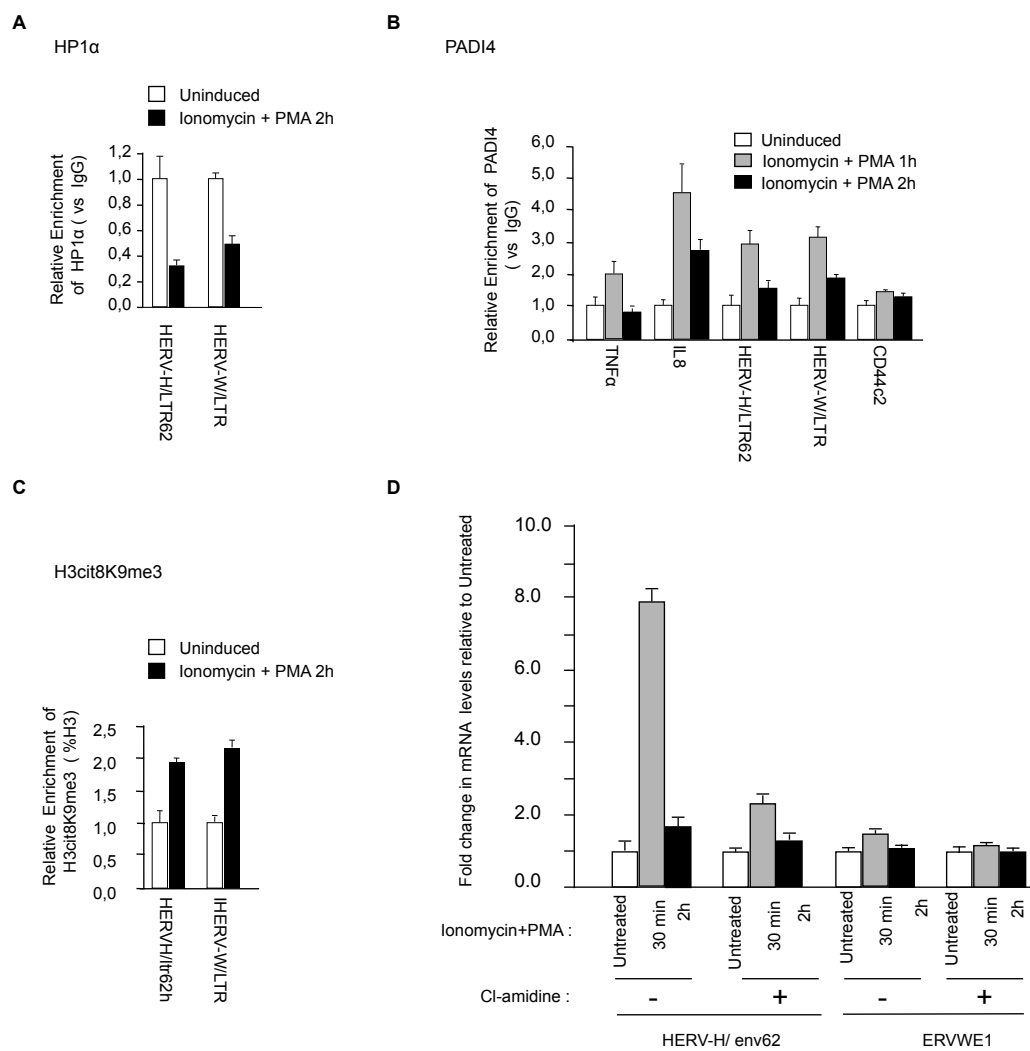

Figure S3 Sharma *et al.*

Supplement: Figure S3 — PADI activity facilitates activation of HERVs in T cells. (A) ChIP with anti-HP1α antibodies was carried out with chromatin prepared from Jurkat cells either untreated or treated with PMA and ionophore (ionomycin). The relative enrichments of HP1α on the indicated LTRs were measured by qPCR. Data are presented relative to non-immune IgG. Changes in enrichment are presented relative to the un-induced control (set to 1). Values are means ± SEM from two PCR measures of two independent ChIP experiments. (B) ChIP with anti-PADI4 antibodies was carried out as in A with indicated time points. Data are presented relative to non-immune IgG. Changes in enrichment are presented relative to the un-induced control (set to 1). Values are means ± SEM from two PCR measures of two independent ChIP experiments. (C) ChIP with anti-H3cit8K9me3 antibodies was carried out as in A. Data are presented as a percentage of histone H3. Changes in enrichment are presented relative to the un-induced control (set to 1). Values are means ± SEM from two PCR measures of two independent ChIP experiments. (D) Total RNA was isolated from Jurkat cells either un-stimulated or treated with ionomycin and PMA minus or plus PADI-inhibitor cl-amidine as indicated. Changes in mRNA levels for the indicated genes were quantified by RT-qPCR. The data are presented as the means ± SEM of duplicate experiments. (PDF) [file pgen.1002934.s003.pdf]

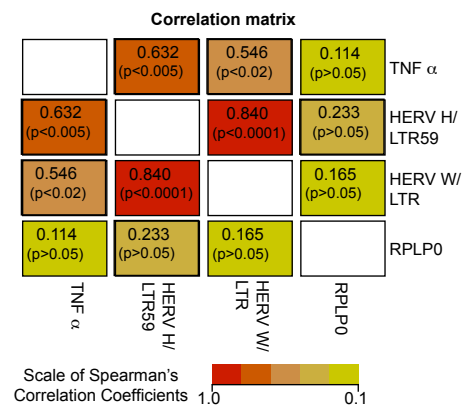

Figure S4 Sharma *et al.*

Supplement: Figure S4 — HP1α in the repression of HERVs and cytokines. Correlation matrix representing the correlation between the presence of HP1α on the promoter regions of RPLP0, TNFα, and HERVs in study individuals analyzed in Figure 6C. Represented values are Spearman Rank correlation coefficients followed by p-values calculated using two sided student t test. p<0.05 was considered as significant. The colour bar at the bottom shows the colour scale according to Spearman's Rank correlation coefficients. (PDF) [file pgen.1002934.s004.pdf]
