# Supplementary material for: Citrullination of Histone H3 Interferes with HP1-Mediated Transcriptional Repression
Source: PLoS Genet. 2012 Sep 13;8(9):e1002934. doi: 10.1371/journal.pgen.1002934 (PMC3441713; doi:10.1371/journal.pgen.1002934)
Supplement: Table S1 — Clinical and demographic data for the lymphocyte samples. The data given for age and duration (years), and EDSS are: mean (range). Abbreviations are as follows: MS: MS patient; U: unaffected relative; PP primary progressive MS; RR: relapsing-remitting MS; SP: secondary progressive MS; M: male; F: female; y: years; EDSS: expanded disability status scale; active: disease course with relapse within a year prior to sampling. None of the patients had diagnosed infections at the time of sampling. (DOC) [file pgen.1002934.s005.doc]

| **ChIP** | | | | |
| --- | --- | --- | --- | --- |
| MS patients | | | | Unaffected relatives |
| *n* | 18 | | | 18 |
| Age (y) | 52 (30-62) | | | 52 (37-69) |
| Gender (F/M) | 11/7 | | | 9/9 |
| MS type | RR | SP |  |  |
| *n* | 10 | 8 |  |  |
| Duration (y) | 14 (4-30) | 18 (11-30) |  |  |
| Age at d. (y) | 31 (19-53) | 36 (24-44) |  |  |
| EDSS | 4 (0-6.5) | 6 (4.5-8.5) |  |  |
| Active (n) | 6 | 1 |  |  |
