# Supplementary material for: Citrullination of Histone H3 Interferes with HP1-Mediated Transcriptional Repression
Source: PLoS Genet. 2012 Sep 13;8(9):e1002934. doi: 10.1371/journal.pgen.1002934 (PMC3441713; doi:10.1371/journal.pgen.1002934)
Supplement: Table S2 — List of primers used in this study. (DOC) [file pgen.1002934.s006.doc]

| **Primers name Sequence (5’ - 3’)** | |
| --- | --- |
| HERV-H/env62 (AJ289709.1) F  R | TGGCCGCTCCTTTGTGTATCTCTT  ACAGGGCAAGTGTCTTCCTAAGCA |
| HERV-H/env59 (AJ289711.1) F  R | TTTATCAGTCCTCCAGGCCCAAGT  AAGAATTGGGAGGACCCAGGACAT |
| HERV-H/env60 (AJ289710.2) F  R | AGGCCTTGACTTACTCACTGCTGA  TTGGAAGGACCCAGGACATCCAAT |
| ERVWE1 (AF072506.2) F  R | TCAGCTTAACTCCCTAGCAGCAGT  TTCGAAGCTCCTCTGCTCTACGTT |
| HERV-K/env102 (AF164610.1) F  R | TGCCAAACCTGAGGAAGAAGGGAT  TGCAGGCATTAAACATCCTGGTGC |
| Sat 2 (AY360176) F  R | CCAGAAGGTAATAAGTGGCACAG  CCCTCCTTGAGCATTCTAACTACC |
| α Sat (M38467) F  R | GAAACACTCTTTCTGCACTACCTG  GGATGGTTCAACACTCTTACATGA |
| TNFα (NM_000594) F  R | GCCCATGTTGTAGCAAACCCTCAA  AGTAGATGAGGTACAGGCCCTCTGAT |
| IL23 (NM_016584) F  R | ACTCAGCAGATTCCAAGCCTCAGT  TGGAGATCTGAGTGCCATCCTTGA |
| IL1A (NM_000575) F  R | AGGAGCTGCCAAGTATTCTGCCAA  AGGGAAGCGCGGGAAACTTATCAA |
| IL16 (NM_172217) F  R | CCAGGAAAGGAAGAAAGGCAGCTT  TGGGCCAAGGAAATCTCAAAGGGA |
| IL8 (NM_000584) F  R | CCACACTGCGCCAACACAGAAATTA  AAACTTCTCCACAACCCTCTGCAC |
| TGFβ1 (NM_000660) F  R | ATGAACCGGCCTTTCCTGCTTC  AAGCAATAGTTGGTGTCCAGGGCT |
| pS2 (NM_003225) F  R | CCAGTGTGCAAATAAGGGCTGCTG  TTCTGGAGGGACGTCGATGGTATT |
| IFIT1 (NM_001548.3) F  R | TATTACCACATGGGCAGACTGGCA  CAGGCCTTGGCCCGTTCATAATTT |
| PADI4 (NM_012387.2) F  R | AAAGTGAGGGTGTTTCAGGCCACA  AAGTCCATGTTGTGCTTTCCACCG |
| RPLP0 (NM_001002.3) F  R | AGGTGTTCGACAATGGCAGCAT  TGCAGACAGACACTGGCAACAT |
| ERVWE1/LTR(AF072506.2) F  R | ACCACATGGCCCAAGATTCCATTC  TCACTTTGGATGTCCGTTCGTGGT |
| HERV-H/LTR62 (AJ289709.1) F  R | CCCGCCAGAGAACAAACTCTCTTT  CCAAATTTCATGCGTGTCTGTGCG |
| HERV-H/LTR59 (AJ289711.1) F  R | ACATATACGCCCAGATGGCCTGAA  ACAGGATGAGCCAGGAGAAGGAAT |
| TNFα promoter(NC_000006.11) F  R | AAACACAGGCCTCAGGACTCAACA  ACCAGGTCTGTGGTCTGTTTCCTT |
| IL8 promoter (NC_000004) F  R | ACTCAGGTTTGCCCTGAGGGGA  TGCCTTATGGAGTGCTCCGGTG |
| pS2 promoter(NC_000021) F  R | AGTACAGTATTTACCCTGGCGGGA  ATGTGGTGAGGTCATCTTGGCTGA |
| GAPDH promoter (NC_000012.11) F  R | CCCGGTTTCTATAAATTGAGCCCGCA  AAAGAAGATGCGGCTGACTGTCG |
| RPLP0 promoter (NC_000012.11) F  R | ACAGAGCGACACTCCGTCTCAAA  ACCTGGCGAGCTCAGCAAACTAAA |
